# Automated classification of time-activity-location patterns for improved estimation of personal exposure to air pollution

**DOI:** 10.1186/s12940-022-00939-8

**Published:** 2022-12-09

**Authors:** Lia Chatzidiakou, Anika Krause, Mike Kellaway, Yiqun Han, Yilin Li, Elizabeth Martin, Frank J. Kelly, Tong Zhu, Benjamin Barratt, Roderic L. Jones

**Affiliations:** 1 Yusuf Hamied Department of Chemistry, University of Cambridge, Lensfield Rd, CB2 1EW Cambridge, UK; 2 Institute for Chemistry, University of Potsdam, Karl-Liebknecht-Straße 24-25, 14476 Potsdam, Germany; 3 Atmospheric Sensors Ltd, SG19 3SH Bedfordshire, UK; 4 Environmental Research Group, MRC Centre for Environment and Health, Imperial College London, W12 0BZ London, UK; 5 BIC-ESAT and SKL-ESPC, College of Environmental Sciences and Engineering, Center for Environment and Health, Peking University, 100871 Beijing, China

**Keywords:** Portable sensor technologies, Multi-pollutant personal exposure, Automated time-activity classification

## Abstract

**Background:**

Air pollution epidemiology has primarily relied on measurements from fixed outdoor air quality monitoring stations to derive population-scale exposure. Characterisation of individual time-activity-location patterns is critical for accurate estimations of personal exposure and dose because pollutant concentrations and inhalation rates vary significantly by location and activity.

**Methods:**

We developed and evaluated an automated model to classify major exposure-related microenvironments (*home, work, other static, in-transit*) and separated them into indoor and outdoor locations, *sleeping activity* and five modes of transport (*walking, cycling, car, bus, metro/train*) with multidisciplinary methods from the fields of movement ecology and artificial intelligence. As input parameters, we used GPS coordinates, accelerometry, and noise, collected at 1 min intervals with a validated Personal Air quality Monitor (PAM) carried by 35 volunteers for one week each. The model classifications were then evaluated against manual time-activity logs kept by participants.

**Results:**

Overall, the model performed reliably in classifying home, work, and other indoor microenvironments (F1-score>0.70) but only moderately well for sleeping and visits to outdoor microenvironments (F1-score=0.57 and 0.3 respectively). Random forest approaches performed very well in classifying modes of transport (F1-score>0.91). We found that the performance of the automated methods significantly surpassed those of manual logs.

**Conclusions:**

Automated models for time-activity classification can markedly improve exposure metrics. Such models can be developed in many programming languages, and if well formulated can have general applicability in large-scale health studies, providing a comprehensive picture of environmental health risks during daily life with readily gathered parameters from smartphone technologies.

**Supplementary Information:**

The online version contains supplementary material available at 10.1186/s12940-022-00939-8.

## Background

Ambient air pollution is a leading environmental risk factor for chronic disease and millions of premature deaths every year worldwide [1]. Much of this evidence comes from epidemiological studies conducted in western countries where networks of outdoor reference monitoring stations have been used to provide indications of the effects of ambient air pollution on population health [2]. Recent studies focused on a global analysis of estimated source contributions to outdoor air pollution and related health effects using updated emissions inventories, satellite and air quality modelling, and relationships between air quality and health at global, regional, country, and metropolitan-area scales [3].

However, as individuals move between different, highly heterogeneous microenvironments that are mainly situated indoors, outdoor static measurements become potentially poor metrics of actual personal exposure [4], leading in many cases to bias and error in health estimations [5]. Adding to the complexity of measuring personal pollutant concentrations, physical activity levels, in turn, affect the dose of inhaled air pollution. For example, while a comprehensive review of the literature found the highest exposure to particulate matter when travelling by car compared with cycling [6], the highest whole trip doses were in fact experienced by cyclists [7] because their higher physical activity levels resulted in greater amounts of pollutant received by the body through larger volumes of inhaled air [8].

Accounting for individual mobility and activity patterns is therefore critical for improved exposure and dose estimations. Such information has been commonly collected with different self-reported questionnaires [9] which often introduce participant error and missing data [10, 11] and increase the participation burden (i.e. time and effort required to complete) [12]. A growing number of studies have taken advantage of increasingly widespread sensor technologies, such as geographical positioning system (GPS) sensors in smartphones, to improve the accuracy of indirect air pollution exposure assessment in large-scale health studies by tracking people’s time-location patterns [13–16].

Time-activity patterns and modes of transport cannot be derived from the GPS raw data directly without further data processing. Only a few studies aim to classify time-activity patterns during daily life using GPS tracking data (smartphone-based or handheld devices), in some cases combined with temperature, light or motion sensors [17–24] to develop primarily rule-based models and/or random forest (RF) learning techniques for a small number of participants over a few days.

In a previous paper [25], we developed, deployed and comprehensively evaluated the performance of a highly portable air pollution sensor platform (PAM) for personal exposure assessments in health studies. We now aim to present a methodological framework as the basis of an approach that automatically classifies and integrates time-activity patterns in personal exposure assessments. This work is toward an overarching aim of capturing total personal multi-pollutant dose in unprecedented detail and, together with medical outcomes, identifying underlying mechanisms of the detrimental effects of specific air pollutants on health. While we use auxiliary parameters collected with a custom-made sensor platform as inputs, such parameters can be readily collected with smartphone technologies, making this method transferable to large-scale health studies.

## Conceptual structure of the time activity model

We developed a model to classify major exposure-relevant microenvironments (*home, work, other static, in transit*) and subclassified them into *indoor* and *outdoor* locations, *sleeping* activities and five modes of transport (*walking, cycling, car, bus, train/metro*) using two open-source software components, R [26, 27] and PostgreSQL [28, 29]. The input parameters for this model (GPS coordinates, noise and accelerometry) were collected with the PAM [25] (S1). Information on data management, post-processing and sensor performance can be found in Chatzidiakou et al., 2019 [25] and in S1.

The PAM has been previously deployed in a number of health studies to monitor the thermal parameters (temperature and RH) and personal exposure of participants to multiple pollutants at high spatial and temporal resolution [30, 31] including carbon monoxide (CO), nitric oxide (NO), nitrogen dioxide (NO$_2$), ozone (O$_3$) and size segregated particulate matter (PM). However, pollutant measurements^1^ and thermal parameters were not used as predictors in this model in order to make this methodology generally applicable to other studies and also transferable to different geographical settings and varying seasons.

**Fig. 1:**
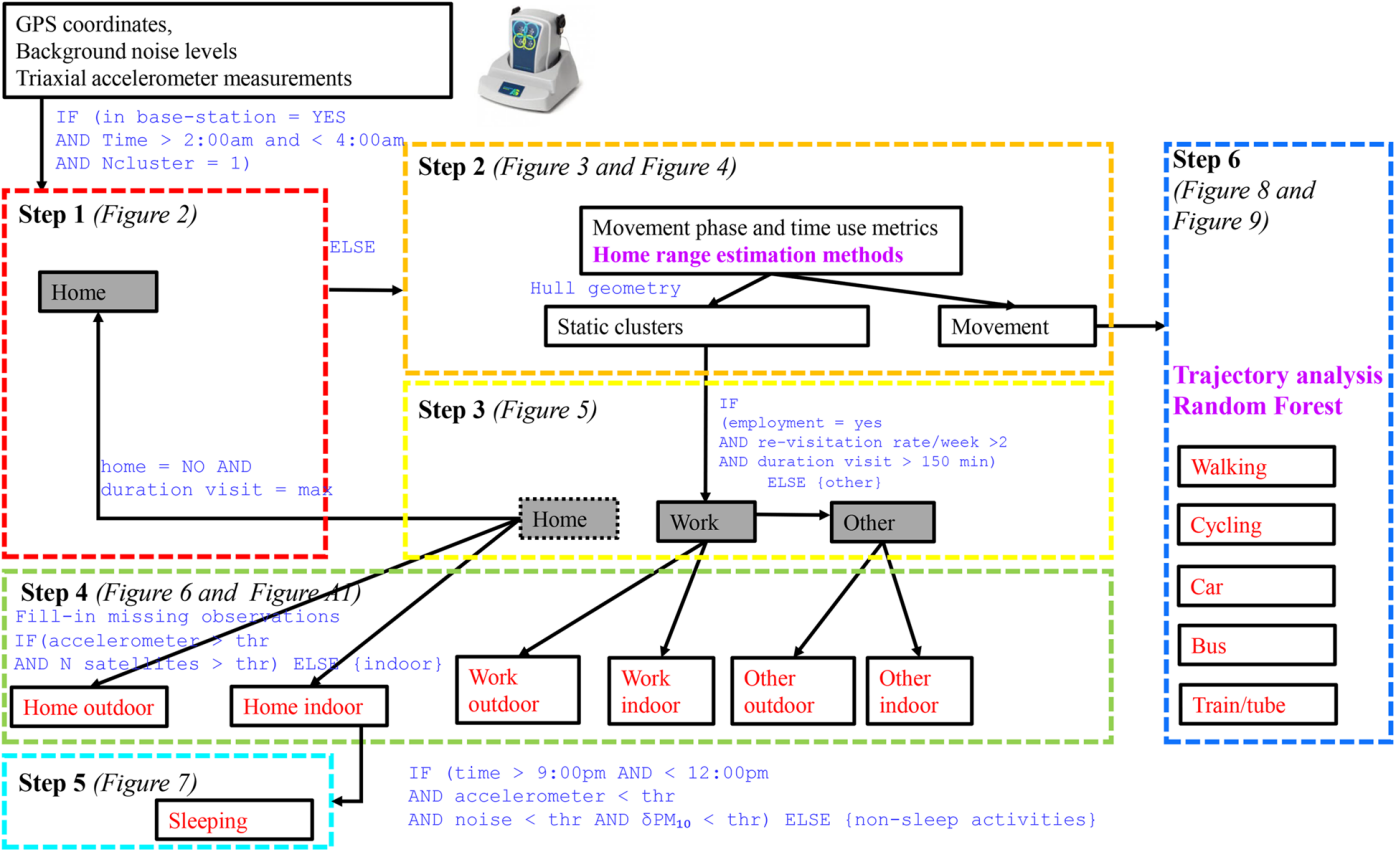
Flow chart of the time activity model

The model can be conceptualised as a series of six consecutive steps, as shown in Fig. 1, to classify major microenvironments, activities and modes of transport (shown in red font), combining rule-based algorithms (blue) and artificial intelligence (AI) methods (purple) summarised in Table 1.

**Table 1:** Summary of AI methods integrated into the time-activity model

AI method	R implementation	Outputs
Home-range method that combines geometric and probabilistic estimators	Time Local Convex Hull (T-LoCoH) [34]	**Polygon (hull) geometry** gives information on directional movement vs. static clusters (*Step 2*). **Visitation rate and duration of visit** enable classifications based on behavioural patterns of the individual (*Step 3*).
Trajectory analysis	Adehabitat LT [37]	Segmentation of movement with the Lavielle method [57]
Predictor selection for Random Forest (RF) classification with three-step elimination process based on data-driven thresholds for high dimensional datasets	VSURF [38]	Predictor variables for RF models collected with the PAM (movement, noise, GPS information) and baseline questionnaire (common modes of transport), and extracted from spatial analysis
RF classification of the mode of transport with the 10-fold evaluation method	RandomForest [65]	Probabilistic classification for each mode of transport

*Step 1* aims to identify the home location with a simple rule-based algorithm to effectively reduce the volume of the data that will be processed with a Lagrangian home-range estimation method [32, 33] in *Steps 2* and *3*. In that way we effectively reduce the volume of data because such methods generally require higher computation power to implement more complex *geometric* or *probabilistic* models^2^. We adopt an existing technique [34] developed in the field of ecology and extend its use to human mobility studies. It combines the robustness of geometric estimators with the simplicity of probabilistic methods to identify important place-marks and fully characterise exposure-relevant behavioural patterns of how the individual uses their activity space.

*Step 4* and *Step 5* employ rule-based algorithms to interpolate missing observations, separate indoor from outdoor static microenvironments and classify sleeping activity. Finally, in *Step 6* we classify modes of transport observations with RF [35], the use of which is considered best practice in travel mode classification [36]. To assist the classification, we perform trajectory analysis [37] to extract useful metrics of movement. Important predictor variables for RF model development were selected with an automated method [38] suitable for high-dimensional data (see Table 1).

Additional to the above main R software environment packages that form the backbone of the model, we used for spatial analysis and visualisation: sp [39, 40], rgdal [41], raster [42], gpclib [43], OpenStreetMap [44], ggplot2 [45] and ggmap [46], rayshader [47]; for time-series analysis, data manipulation and visualisation: openair [48], dplyr [49], plot3D [50]; and for clustering and classification: caret [51], dbscan [52].

The model development steps are described in detail below and illustrated using information from one representative participant over a period of one week.

### Step 1: Rule-based algorithm for home location identification to reduce computational demand of the time-activity model

**Fig. 2:**
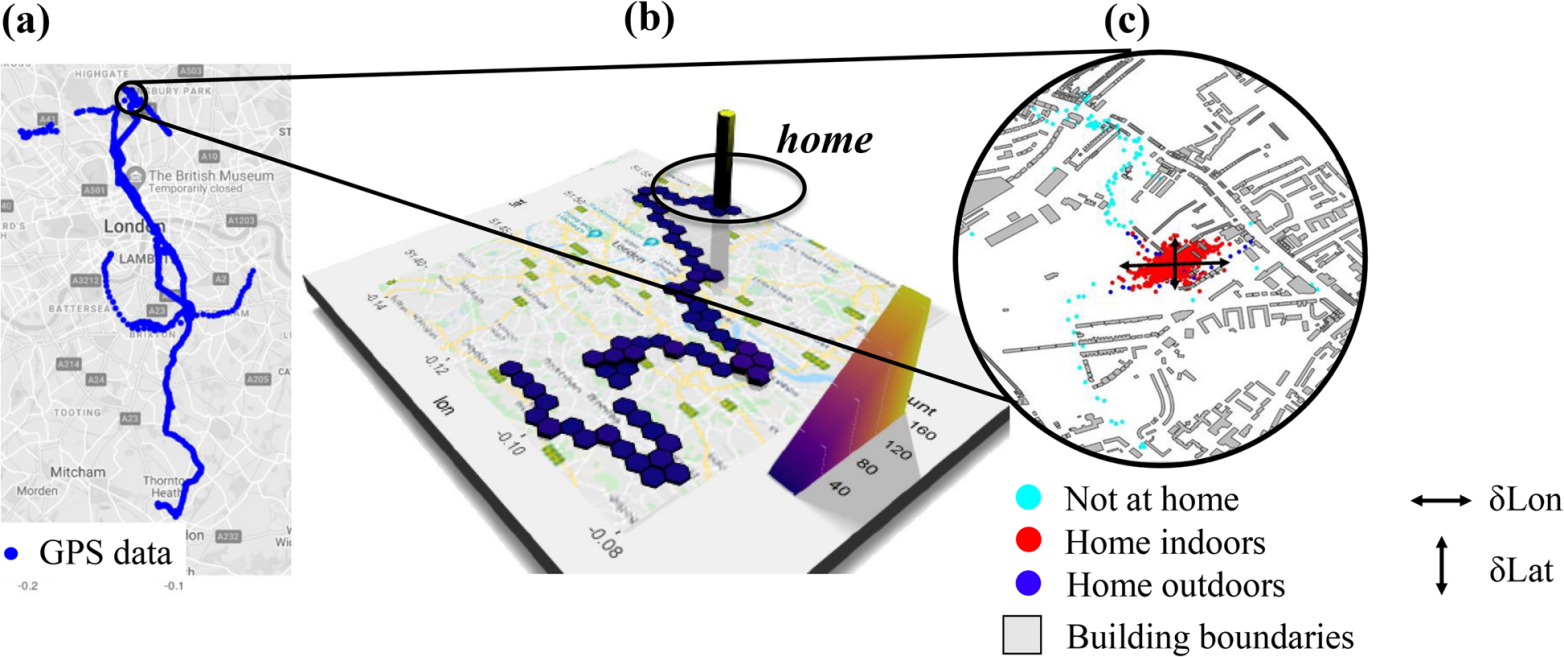
Graphical flow chart of home identification of the time-activity model. (a) Map of the raw GPS data (blue) collected from a representative participant carrying a personal air quality monitor over a week. (b) 3D density plot of participant’s time budget projected on a map. “Home” location has the highest point density (i.e. most time spent). (c) A spatial elliptical zone created with a rule-based model to identify “home” that included indoor (red) and outdoor (blue) micro-environments (separated in Step 4). The spread distances ($\delta$Lon and $\delta$Lat) around the centroid are often larger than the GIS footprints of the buildings (grey) and depend on multiple factors. Map data from Google Maps 2021 (a and b) and OpenStreetMap(c)

The rationale of this simple algorithm relies on common behavioural patterns of most people in western settings, who tend to spend most of their nighttime at home (Fig. 2b). This assumption holds particularly in this study but it can be readily adjusted to shift workers who may be at home at different times. We identified periods when the PAM was in the base-station - the dock used by participants to charge the PAM at home - (as indicated by the input voltage of the unit) and when the local time was between 02:00-04:00 AM; therefore, making it more likely that the participant was at home. Due to GPS errors, these points tended to be displaced around the home location as illustrated in Fig. 2c, often falling outside the GIS building boundaries.

A clustering algorithm (in this case k-means in R) was applied to this data subset to determine whether the scattered points formed a single cluster for each participant. For a few participants, multiple clusters were detected hence *home* could not be determined in this step (for example, due to sleeping in multiple locations or lack of satellite reception during the selected period) and for these participants *home* was subsequently classified in *Step 2* as the location where the participant spent most of their time.

If a single cluster was identified, a spatial elliptical zone (*“buffer zone”*) was created around each home microenvironment by extracting the centroid coordinates and the individual spread distances ($\delta$Lon and $\delta$Lat) (Fig. 2c). Any spread is expected to depend on contextual factors (such as building construction characteristics and GPS signal quality) and was typically found to range from 60m to 500m( [23, 24]. Data points within that spatial zone (Fig. 2c) were classified as *home* and were separated into *indoor* and *outdoor* in *Step 4*.

### Step 2: Stationary locations and movement patterns from space-use metrics

**Fig. 3:**
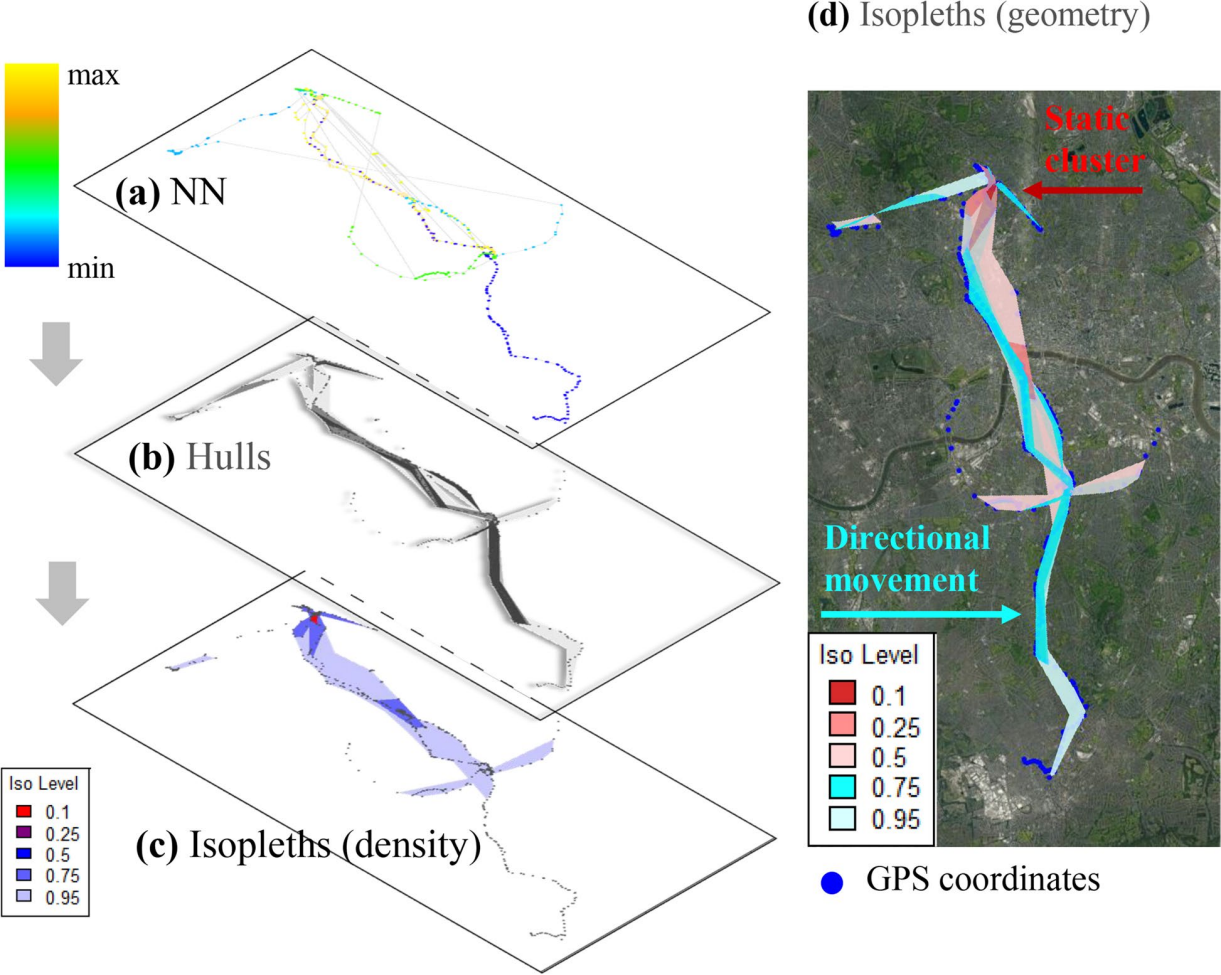
Example graphical flow of space-time utilisation distribution analysis (step 2) implemented with the T-LocoH package in R. (a) First, nearest neighbours were identified with the adaptive method ($\alpha$-NN) (b) Minimum convex polygons (hulls) were then produced from these $\alpha$-NN (c) Hulls were merged by point density to create density isopleths (utilisation distributions) to characterise space intensity use. (d) Hulls were merged by the eccentricity of the bounding ellipse to create elongation isopleths to characterise movement and were projected on a map (Google Maps 2021)

The remaining observations (i.e. those not belonging within the home spatial zone) were analysed with the R package T-LoCoH [34] (Table 1) to distinguish between movement and static activities. The strength of this technique is that it models space-use (*Step 2*) and time-use (*Step 3*) simultaneously. It does that by employing a scaling that relates distance and time in reference to an individual’s characteristic velocity (time-scaled distance). Previous studies have found that such estimators that incorporate a temporal component with individual-specific parameters generally perform better than traditional estimators [53]. We first used the extracted geometric features to classify static clusters and directional movement following the workflow illustrated in Fig. 3 and described below:

- **Figure **3**a: Defining nearest neighbours with the adaptive method**. GPS data were first converted to a conformal (Universal Transverse Mercator) projection because it preserves local angles and represents shapes accurately and without distortion for small areas. The algorithm begins by identifying a set of nearest neighbours around each point (Fig. 3a) based on their time-scaled distance. Participants did not utilise areas in a uniform pattern, but rather selected areas based on their individual activities, resulting in heterogeneous coverage of both dense and sparse areas. To account for these patterns, the selection of nearest neighbours [34] was performed with the adaptive method ($\alpha$-NN).^3^
- **Figure **3**b: Geometry of the enclosing polygons**. Each parent point and its nearest neighbours were bound together with a minimum convex polygon or a hull (Fig. 3b). Hulls are the building blocks of the subsequent analysis and have different properties (point density and shape) which in turn provide important information on the use of space. The eccentricity of the ellipse bounding a hull is a good approximation of its shape, which specifies whether an individual is in movement or stationary. For example, a bounding ellipse with an eccentricity value close to zero resembles a circle and indicates areas where the individual was stationary for an extended period, resulting in a dense cluster of points similar to the red cluster presented earlier in Fig. 2c. In contrast, elongated bounding ellipses have an eccentricity value close to one because they enclose nearest neighbours that form linear segments indicating areas of directional movement.
- **Figure **3**c and d: Defining areas with similar polygon geometry**. Depending on the research question, hulls can be sorted by a selected property, and then merged together to form isopleths that connect areas with the same numerical value of that property. In the example of Fig. 3c, areas that are used by the participant with the same intensity were merged to produce traditional utilisation distributions. When hulls with similar eccentricity values are merged as shown in Fig. 3d, similar movement patterns are connected in a single isopleth ranging from the highest elongation hull value close to 1 (cyan) capturing points in movement to the lowest elongation value close to 0 (red) indicating dense clusters of GPS points. In this way, similar movement patterns are grouped into a single isopleth. Isopleths typically contain 95% of the total points excluding outliers that occur frequently and could skew the results [34].

**Fig. 4:**
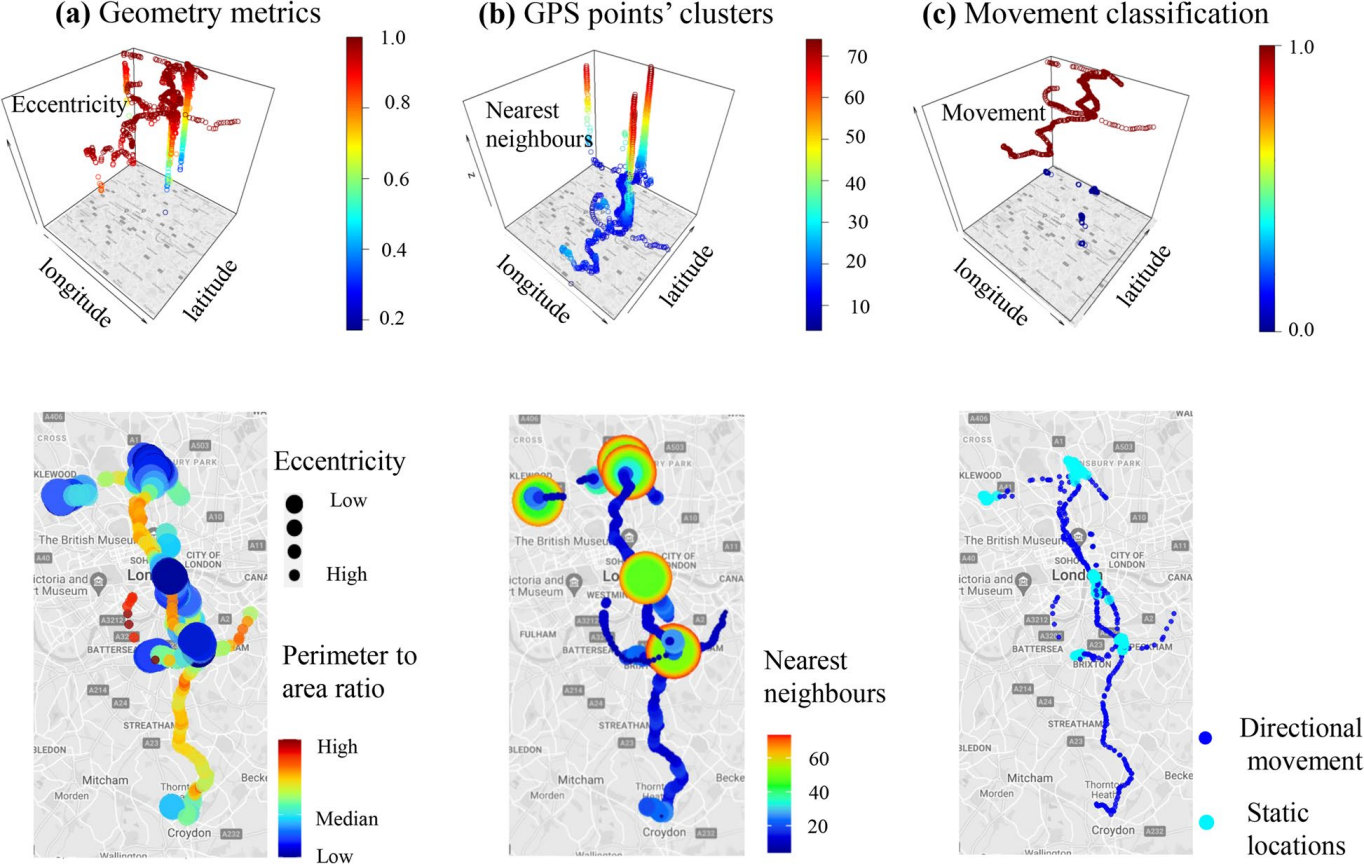
Selected features for the classification of static clusters and directional movement are shown in 3D (top) and projected on maps (bottom) in a colour and size-scale. (a) The eccentricity and the perimeter-to-area ratio of the enclosing ellipse provide information on hull geometry and directional movement. (b) Dense clusters of nearest neighbours were constructed in areas used more frequently. (c) Final classification of static clusters and in-movement location based on thresholds of these features

Figure 4 illustrates these extracted geometric features in 3D (top) and 2D (bottom) maps. The graphs show that both the eccentricity of the enclosing ellipses (Fig. 4a) and the number of nearest neighbours (Fig. 4b) provide strong discriminatory power to separate directional movement from static locations (Fig. 4c) with suitable thresholds.

### Step 3: Behavioural patterns from time-use metrics

In the previous step, we constructed hulls using the time-scaled distance between GPS points. The time-scaled distance distinguishes points that are far away in time even though they may be close in Euclidean space. Therefore, the hulls are local not only in space but also in time enabling the characterisation of behavioural patterns with two important temporal features: the duration of visit and the revisitation rate over 12 hours to capture diurnal patterns of human behaviour.

**Fig. 5:**
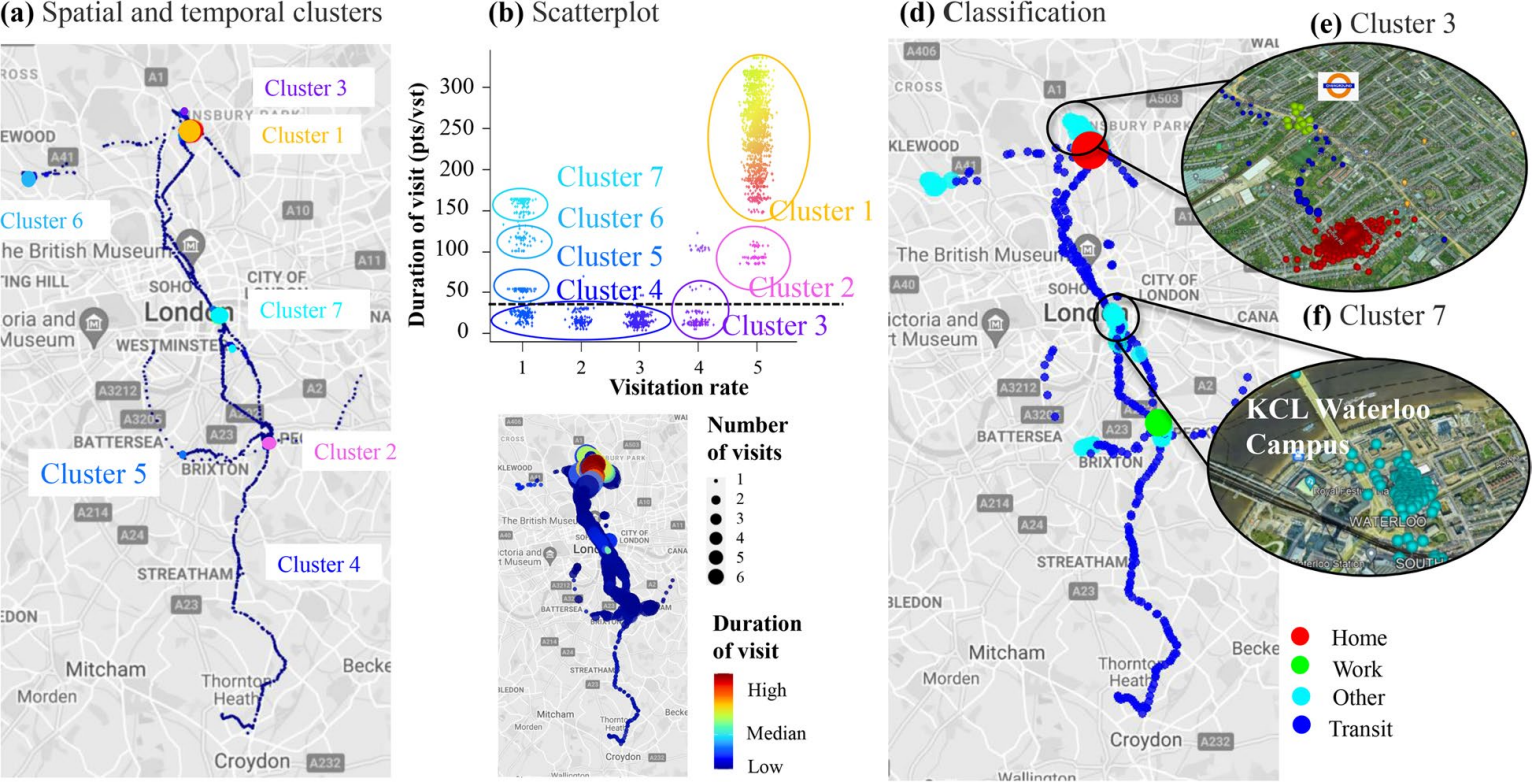
Flow chart of the time activity model (a) Map of seven distinct clusters identified based on temporal information contained in the isopleths. (b) Scatterplot of the visitation rate (over 12h) vs the duration of visit (average points per visit). The dashed black line indicates the threshold in the duration of visit that discriminates between static locations from directional movement. (c) Map of time-use metrics during the participation week. The colour scale indicates the total minutes spent in each location while the size of the points corresponds to the number of visits. (d) Final classification of static locations into three microenvironments (“home”, “work”, “other”) and in movement based on spatiotemporal behavioural patterns of the individual. (e and f) Subclassifications of “other” visited microenvironments derived from GIS information and behavioural patterns

The scatterplot of Fig. 5b shows that, based on the revisitation rate and duration of visit, seven distinct clusters were identified and projected on a map in Fig. 5a. Overall, three main categories can be identified: clusters which were visited often and for extended time periods (*Clusters 1* and *2*), clusters where the participant spent limited time (*Clusters 3* and *4*), and finally clusters visited once during the week but for longer time (i.e. more than an hour as in *Clusters 4, 5, 6* and *7*).

These extracted time-use metrics assisted the automated classification. *Cluster 1* (Fig. 5b) could be classified as *home* (if it had not been classified as such in *Step 1*) as shown in Fig. 5d. The cluster visited frequently and for extended time periods and was classified as *work* (in this example *Cluster 2*).

*Cluster 4* was classified as in-movement, not only based on the hull metrics in *Step 2*, but also based on the low duration of visit as shown in Fig. 5b. Within *Cluster 4*, differences in revisitation rates (as illustrated by the size of points in Fig. 5c) can be used to distinguish daily commuting routes. For example, points between *home* and *work* have been revisited 3 times compared with points south of *work* that have only been visited once.

Finally, details on locations visited for extended periods but less often, (*Clusters 3,5,6* and *7*) could be retrieved from GIS maps and common behavioural patterns. For example, *Cluster 3* in proximity to home had short but frequent visits within the spatial zone of the overground station and could be classified as *waiting for the train* (Fig. 5e). Contrary, *Cluster 7* was only visited once but had a high duration of visit and together with the GIS information could have been classified as a secondary workplace location (Fig. 5f, KCL Waterloo Campus) .Both subclassifications were confirmed by the manual diary entries. Although this approach shows the capabilities of the model, it is beyond the scope of this work to subclassify each microenvironment and they were, therefore, all grouped as *other* but with a unique identifier (Fig. 5d). Currently, services such as Google Places API have the ability to return information on places of interest.

Overall, the technique illustrated here provides a simultaneous analysis of spatial and temporal patterns to separate static locations from directional movement and infer behavioural patterns on the use of space of the individual.

### Step 4: Separating indoor from outdoor microenvironments

GPS signal loss is common in indoor microenvironments, such as in the underground metro system, in urban areas with tall buildings and structures, or when the monitor is static in an indoor microenvironment for extended periods. In such cases, a large percentage of geo-coordinated observations may be missing. While this percentage will vary between deployments, in our sample it was found to be $\sim$ 40%. A rule-based algorithm was developed to interpolate the missing locations using previous- and last-known locations and PAM auxiliary parameters as inputs (S2, Fig. A1), and in this way classify indoor microenvironments with limited GPS satellite reception.

**Fig. 6:**
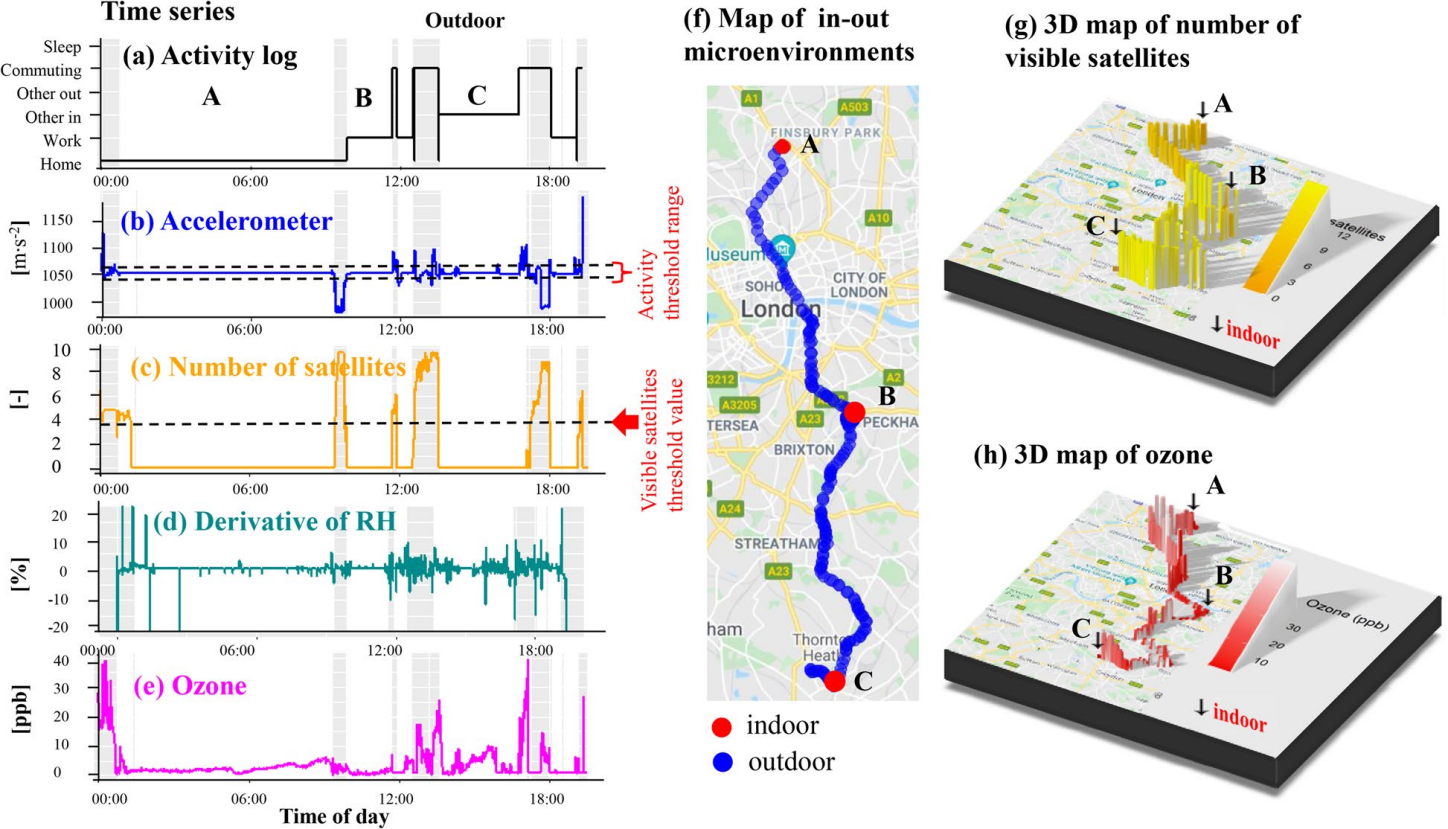
Identifying transitions between indoor and outdoor microenvironments. (a) Time series of manual activity logs. Grey shaded areas indicate periods flagged as outdoor microenvironments with the rule-based algorithm. (b and c) Participant-specific thresholds (black dashed lines) of two parameters collected with the PAM (acceleration and number of visible satellites) were used to flag transitions between microenvironments. (d and e) In addition to manual logs, sudden changes in RH and ozone levels were used to evaluate the performance of the algorithm indirectly (f) Corresponding map of indoor (red) and outdoor (blue) microenvironments classified with the rule-based algorithm (g) 3D map visualising the number of satellites transmitting to the PAM GPS receiver. (h) 3D map of PAM ozone levels

Once missing observations were largely accounted for, each static microenvironment (*home, work, other*) was classified as *indoor* or *outdoor* with a rule-based algorithm (Fig. 1) formulated on the hypothesis that abrupt changes in acceleration and GPS signal quality are indicative of transitions between microenvironments. The algorithm used participant-specific thresholds of these two parameters to classify indoor and outdoor microenvironments and is visualised in Fig. 6 using data from a single participant-day.

Figure 6 presents the time-series of selected parameters (acceleration, number of satellites) to develop the indoor-outdoor separation algorithm (Fig. 6b and c), the corresponding map (Fig. 6f) with indoor (red) and outdoor (blue) classifications, as well as a 3D map of the number of satellites transmitting to the PAM receiver (Fig. 6g). Higher numbers of satellites are typically seen outdoors due to signal blockage in indoor environments (Fig. 6c and g).

We have included the manual diary logs, ozone levels measured with the PAM (Fig. 6e and h) and the time-derivative of RH as indirect ways to confirm the performance of the algorithm. During daytime, ozone levels are consistently very low indoors as shown in the 3D map in Fig. 6h (for example, locations A, B and C) due to the high reactivity and depletion on indoor surfaces, the limited solar radiation and the lack of indoor sources [54]. They are also significantly reduced during certain modes of transport (for example, B to C) for similar reasons. Finally, we have previously shown in a controlled experiment that fast changes in RH can flag rapid environmental changes as a person moves between different microenvironments [25]. Therefore, the time-derivative of RH could be used to flag the indoor-outdoor transition with high time precision (Fig. 6d).

The evaluation of the model with a single participant-day so far shows a high level of agreement between the algorithm predictions (grey shaded areas) and the manual activity logs (black line) shown in Fig. 6a. Additionally, the sharp spikes in the derivative of RH (Fig. 6d), and the rapid changes in ozone concentrations (Fig. 6e) further support that the rule-based model can discriminate between indoor and outdoor microenvironments well. Full evaluation is presented in Section 3.

### Step 5: Characterisation of sleeping activity

**Fig. 7:**
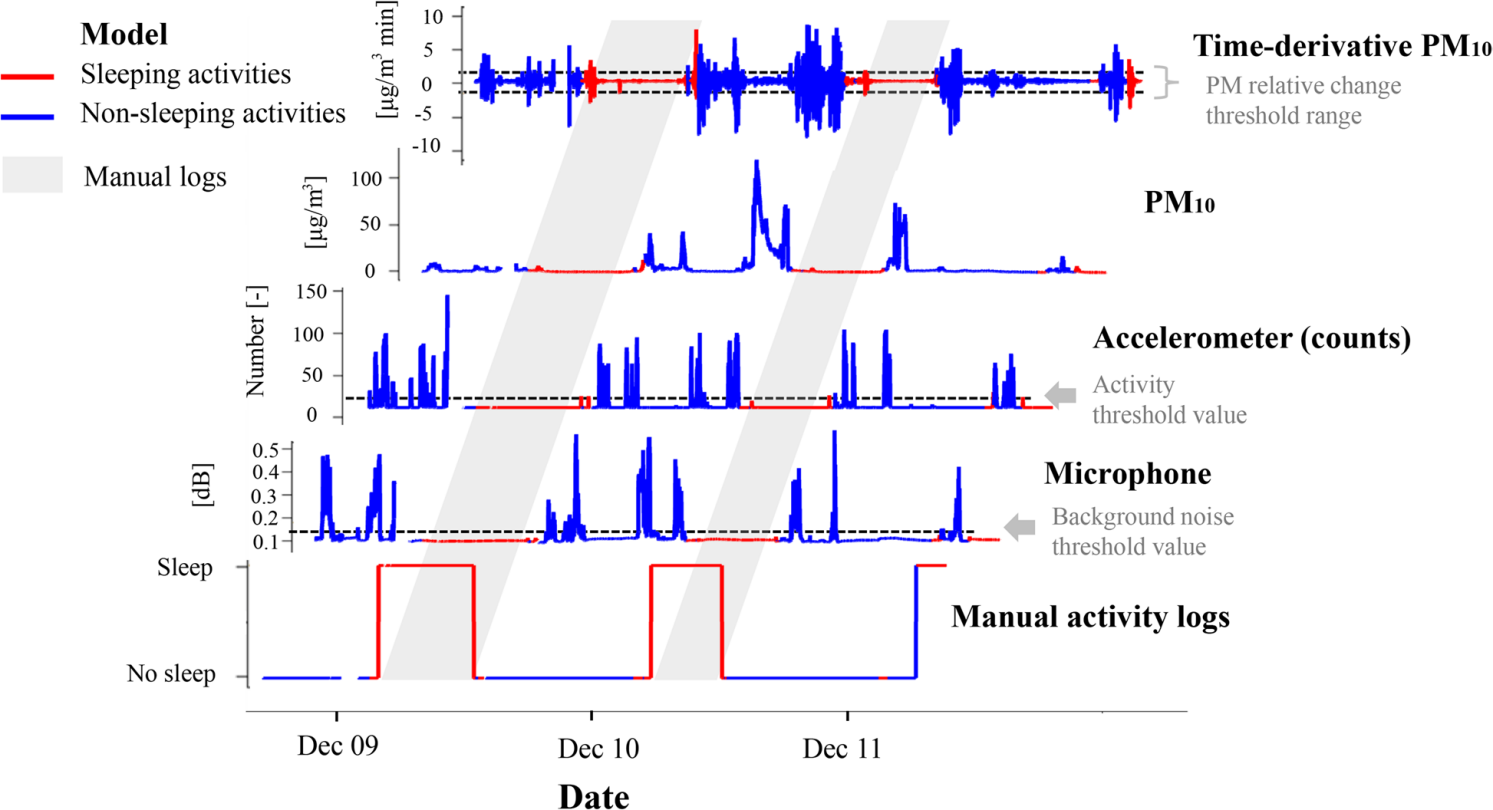
Illustrative time series waterfall plot of selected PAM parameters used to classify sleep activity with a rule-based algorithm. Participant-specific thresholds (black dashed lines) were set for microphone and accelerometer levels and for the time-derivative of PM$_{10}$. Red line segments show time periods that the model classified as “sleep” while the blue line segments indicate non-sleep activities. Manual activity logs are presented for comparison as a time-series and as a grey shaded area

The indoor home microenvironment was subdivided into *sleep* and non-sleep periods with a rule-based model (Fig. 1) based on the hypothesis that participants sleep when background noise levels and movement are the lowest. Additionally to the accelerometer showing that the PAM was stationary (Fig. 7), relative changes in the larger fractions of particulate matter were used as an indicator of movement in the room because larger particles would be expected to resuspend during periods of physical activity of the occupants [55]. The time derivative of PM$_{10}$ was used to detect these changes of concentrations (Fig. 7). While in this case we use a specialised optical particle counter, such information on participant movement could have been collected with widely used wearable sensors (such as smartwatches). Participant-specific statistical thresholds were set for these three parameters to detect sleep activities followed by a smoothing filter over a 10 min rolling window applied on the binary classification to remove small disruptions.

Figure 7 shows that in this example there is an excellent agreement between manual activity logs (grey shaded area projected from time series) and algorithm-based classification (line segments highlighted in red) with a marginal overprediction of sleep because the algorithm cannot separate downtime before sleep from actual sleeping activity as recorded in the diary. This rule-based model for sleep is evaluated using the whole dataset in Section 3.

### Step 6: Classification of transit modes

The periods classified as *in transit* were classified into, in this case, five modes of transportation. First, we created and selected predictor variables for the RF models which were trained and evaluated with a k-fold method as described below:

#### Trajectory analysis and segmentation

**Fig. 8:**
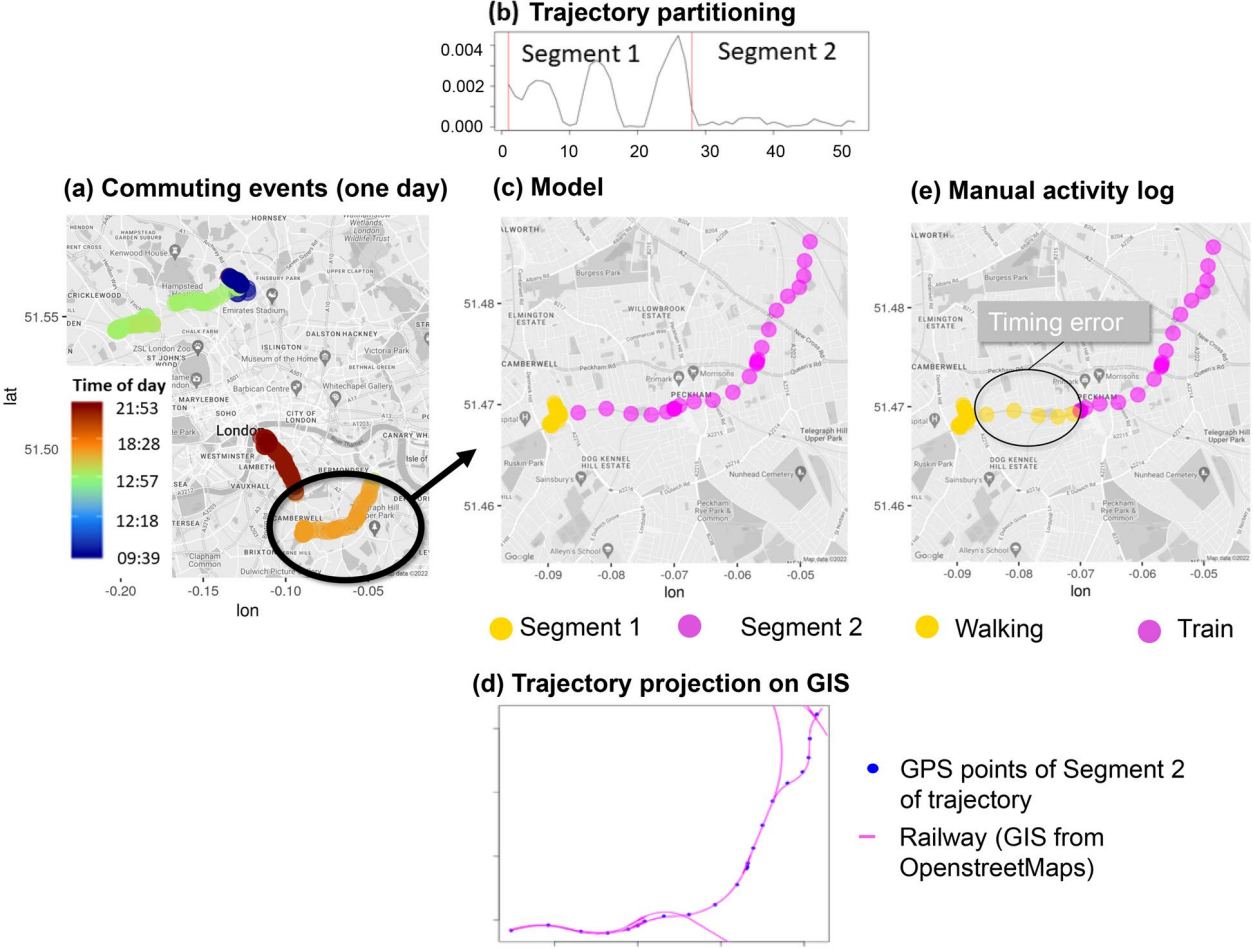
Flow diagram of movement analysis implemented in adehabitat LT package in R. (a) Map of commuting events (journeys) of one participant during a typical day. The colour scheme indicates the time of day. (b) Segmentation of one trajectory (journey 18:28 in orange in a) using the Lavielle method identified two segments in the data. (c) The corresponding map of the trajectory in colour scale to differentiate the two segments. (d) Projection of segment 2 on the GIS system retrieved from Openstreetmap. The GPS points (blue) overlap with the railway infrastructure shown in magenta. (e) Corresponding map of the participant manual diary logs of that journey (see subsection 3.1). Visual inspection shows a delay in diary input that would result in small errors in model evaluation

In-transit observations for each participant were grouped into individual commuting events (journeys). Stops were part of a journey if the participant stayed in a static location for less than 20 min (see Fig. 8a, otherwise a new journey was defined). Each journey was assigned to a “regular trajectory” [56] i.e., a continuous curve connecting successive locations of an individual recorded at regular intervals.

During a single journey, people are likely to change their mode of transport (for example, walking to the metro and then taking the train). To account for that, each trajectory was partitioned into smaller segments based on changes in patterns of movement data with the Lavielle method [57] implemented in the adehabitat LT package in R [37]. To illustrate this method, one journey is selected as a case study, partitioned automatically into two segments (Fig. 8b). These two segments of the trajectory are plotted on a map (Fig. 8c) by colour and projected on GIS (Fig. 8d) to retrieve information on public transport infrastructure and road networks. Because the points of the second segment fall on the railway network (magenta line in Fig. 8d), Segment 2 corresponds to a train ride. Manual activity logs of the participant are presented in Fig. 8e where a timing error in the activity entry in the transition between *walking* and *train* is indicated by both the GIS information and the speed derived from the distance between successive points.

#### Variable selection for RF

After all participant trajectories were segmented and projected on the GIS system, we had 60 variables that could be potentially used as predictors for the classification:

- 31 variables collected with the PAM: hour of the day, GPS coordinates and GPS diagnostic information (i.e., visible satellites), and extracted features from the accelerometer and microphone measurements which could have been collected with a smartphone (See full list Additional files, Table A1).
- 3 variables collected with the questionnaire: car and bicycle ownership and frequency of public transport use.
- 19 movement-phase metrics: Extracted with spatio-temporal clustering and trajectory analysis including absolute and relative angle of movement, Euclidean distance between consecutive points (speed), PAR of hulls etc. (See full list Additional files, Table A2)
- 7 variables retrieved from projecting the data on GIS: highway, railway, sidewalk, cycleway, busway and bus and train stops.

**Fig. 9:**
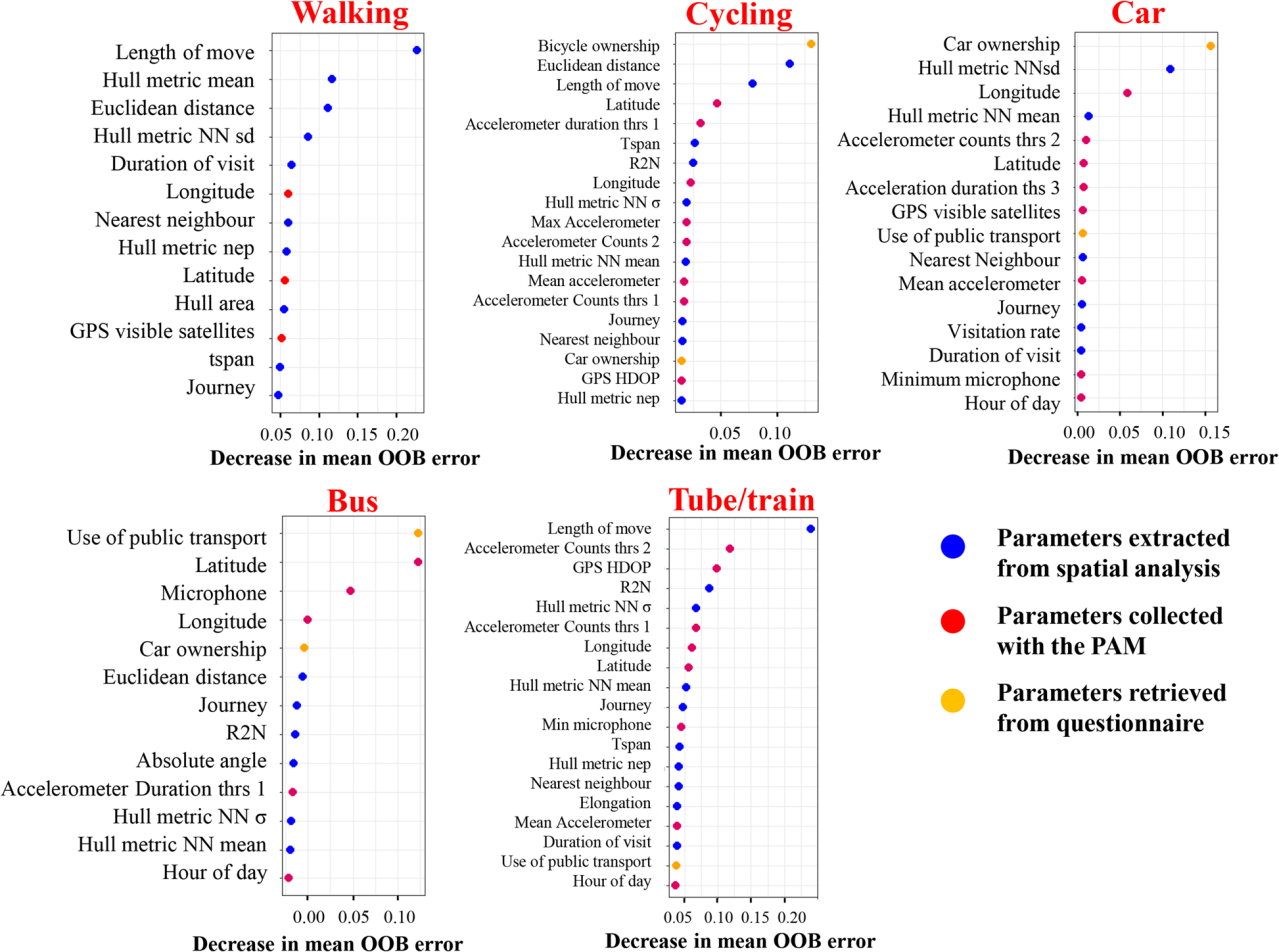
Variable importance plots selected with the VSURF package in R for each mode of transport

Variable selection for the classification was implemented using RF in the VSURF package [38] in R which is suitable for high dimensional datasets. This strategy does not depend on specific model hypotheses but is based on data-driven thresholds to make decisions. VSURF successively eliminates predictor variables in three steps: (1) starting with the preliminary elimination and ranking where all 60 variables were ranked by sorting the score of Variable Importance (VI) averaged over 50 RF runs. (2) In the second step, a nested collection of RF was constructed to select variables that led to the smallest out-of-the-bag (OOB) error. (3) Among those retained in the previous step, final variables for prediction were selected by constructing an ascending sequence of RF models and testing the variables in a stepwise manner. A variable was retained only if the decreased OOB error was significantly greater than the average variation obtained by adding noisy variables (Fig. 9)(calculated threshold here = 0.01).

The most important predictor variables retained with this method make intuitive sense: for *walking* and *train* the most important predictor was distance travelled, for *cycling* and driving it was the ownership of a bike and a car respectively, while for the *bus* it was the use of public transport (Fig. 9). This indicates that an equally valid approach would be to manually select and evaluate predictor variables based both on data-driven thresholds and hypothesis testing. Finally, we found that parameters extracted from GPS data with spatial and movement analysis methods (T-LOCOH and adehabitat LT) were more important predictors than raw PAM variables stressing the importance of appropriate feature extraction to optimise machine learning techniques.

#### RF development

Sensitivity tests were conducted for determining the maximum tree depth and number of trees. The RF was evaluated with a k-fold cross-validation method [58], which is a robust method for estimating the accuracy of a model. The dataset was split randomly into 10 mutually exclusive datasets of equal size. Then, on each iteration a new RF was trained independently on 9 subsets and evaluated on the remaining 1 subset of data, and this procedure was repeated 10 times. The final prediction error rate was calculated as the average performance metric of the 10 models. The advantage of this method is that all observations are used for both training and validation, and each observation is used for validation exactly once.

## Evaluation of the time activity model

This section firstly describes the participant sample and recruitment procedures before comparing manual activity logs with model classifications.

### Collection of activity logs for time-activity model evaluation

A convenience sample of 37 participants (office workers) were recruited (Additional Files, Fig. A2) via email lists and other methods. Participants were recruited from London, a megacity population $\sim$9M and Cambridge, a relatively small UK city population $\sim$125K, to allow evaluation of the model in different urban settings. One London and one Cambridge participant were excluded from the analysis due to incomplete diary entries ( < 24h).

Upon enrolment, participants were briefed on the aims of the study, gave informed consent and filled in a standardised questionnaire of baseline information on exposure-relevant lifestyle (including e.g. car ownership), personal and demographic factors. The age distribution of the 35 participants ranged from 18 to 65 years, and were all in employment (Additional Files, Table A3).

Each participant was provided with a PAM [25] and was asked to carry it for at least one week typical of their normal activities.The average deployment time was 9 days with a minimum of 3 and a maximum of 20 days. Participants were informed that the monitors utilised GPS technology and were reassured that this information would not be accessed in real-time, but only used at the end of the study to analyse overall spatial and temporal relationships of anonymised data. No action was required by the participants to operate the PAM, other than to place it in its base-station overnight for charging and data transmission [25].

While carrying the PAM, they were asked to keep activity diaries using commercial smartphone apps [59, 60]. Smartphones were provided on request. The time-activity diary was semi-structured with some initial activities inserted in the diary as an example (e.g. *“sleeping”*). Participants were encouraged to fill in additional activities according to their lifestyles. At the end of the study, diary entries of the time-activity-location patterns were retrieved from their smartphones. Other than a personalised report of their own exposure profiles as feedback (see example Additional Files, Fig. A3), they did not receive compensation for their participation.

Overall, the participants reported 665 time-activity entries. These entries were assigned to two core categories: *location* and *activity*. Classifications were derived from the diaries by grouping similar entries together (e.g. supermarket, grocery, food shopping). Three exposure-related classifications were developed for the category location and eight classifications for activity (Additional Files, Table A4). These were integrated into the measurement dataset by labelling each data point of the time series with a numerical classifier. Activity logs were checked manually to identify periods of obviously erroneous entries, such as (a) being at two locations simultaneously; or (b) contradictory activities (e.g., *sleeping* and *cycling*) which were removed ($\sim$ 5% of the activity logs).

### Aggregated participants’ time budgets

Over 1.26M observations of PAM measurements at 20 sec time resolution were retained for the analysis (data capture rate 85%) and were averaged over 1-minute, resulting in N$_{obs}$ $\sim$422K of which $\sim$91% had an associated manual log.

**Fig. 10:**
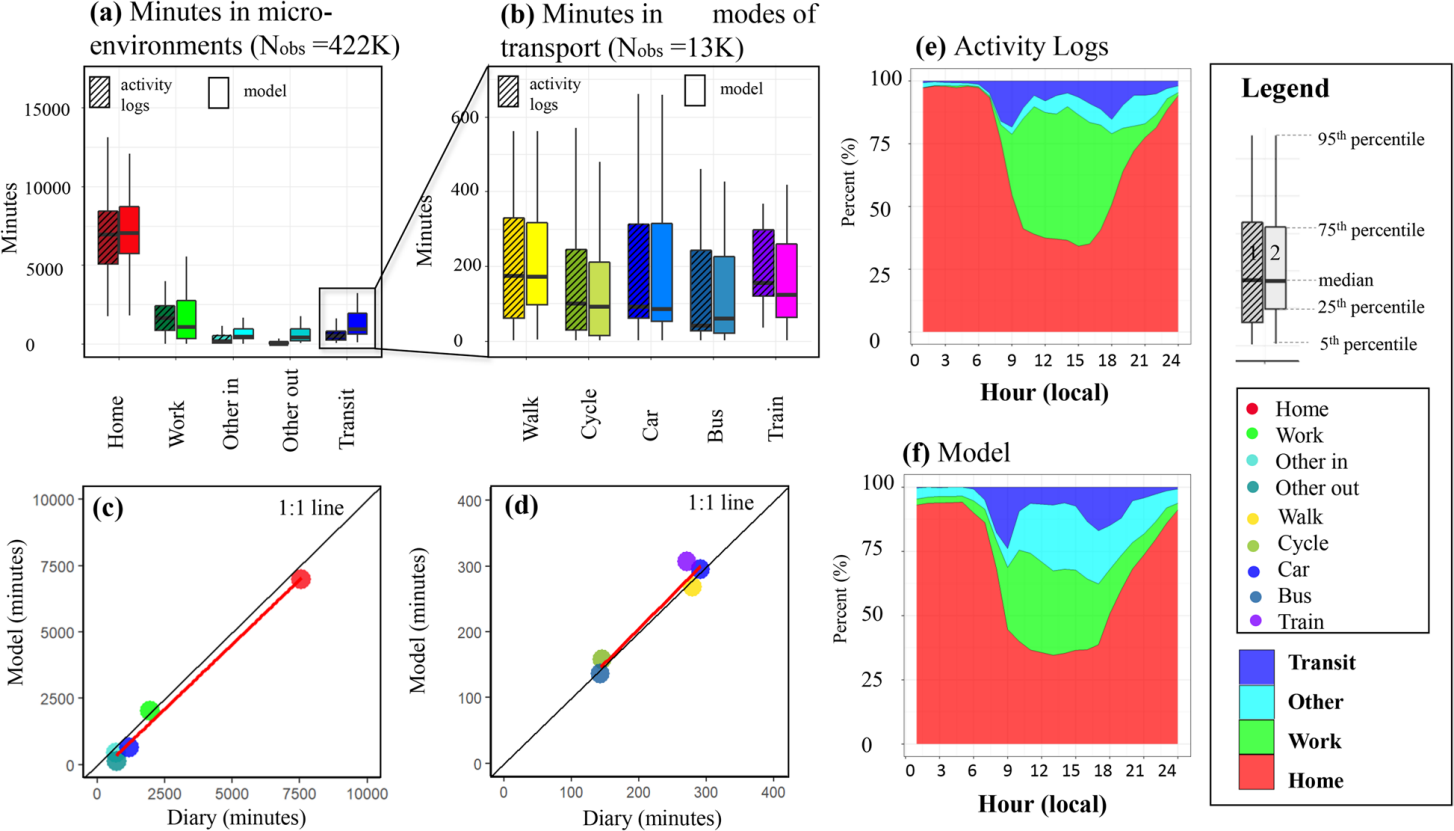
Participants’ time budgets. (a and b) Boxplots of participants’ time budgets in different static microenvironments and modes of transport classified with activity logs (left, shaded boxplot) and the model (right, solid-colour boxplot). (c and d) Corresponding scatterplots of mean time (in minutes) spent in visited microenvironments are shown in a colour scale at the bottom. (e and f) Average diurnal time budget profile of all participants classified with the activity logs and with the model

The aggregated time budgets and diurnal time-activity patterns of the participants are shown in Fig. 10. Average minutes per day spent in different microenvironments and modes of transport classified with the model show an excellent agreement with the activity logs (Fig. 10a-b), with strong linear correlation (Fig. 10c-d). In this study, the participants spent most of their time indoors at home (59.2%, min-max: 29.1%- 89.4%) or at work (16.2%, min-max: 0.0%- 41.2%), together accounting on average   75.4% of the total time budget. Time spent in other indoor static locations accounted for 9.3% (min-max: 0.0%-31.3%). Visits to outdoor microenvironments occupied only a small portion of the participants’ time budget at 0.4% (min-max: 0.0%-3.9%). Travelling accounted for 5.2%, (min: 0.1% - 11.8%).

The diurnal time budget aggregated among all participants captured by the model (Fig. 10f) agreed with the manual activity logs (Fig. 10e). The model overpredicted *other static* but underpredicted *work* possibly because participants had multiple work microenvironments but the model classified only the primary cluster as work (visited often and for extended time periods) as shown in *Step 3*. Regardless, the model managed to capture the participants’ time-activity patterns well. Their patterns followed wider socio-economic patterns of adults in employment with distinctive commuting events during “rush hour” at 9:00 am and after 5:00 pm when participants returned home and stayed there until 6:00 am (Fig. 10f).

### Evaluation of the time-activity model with confusion matrices

**Fig. 11:**
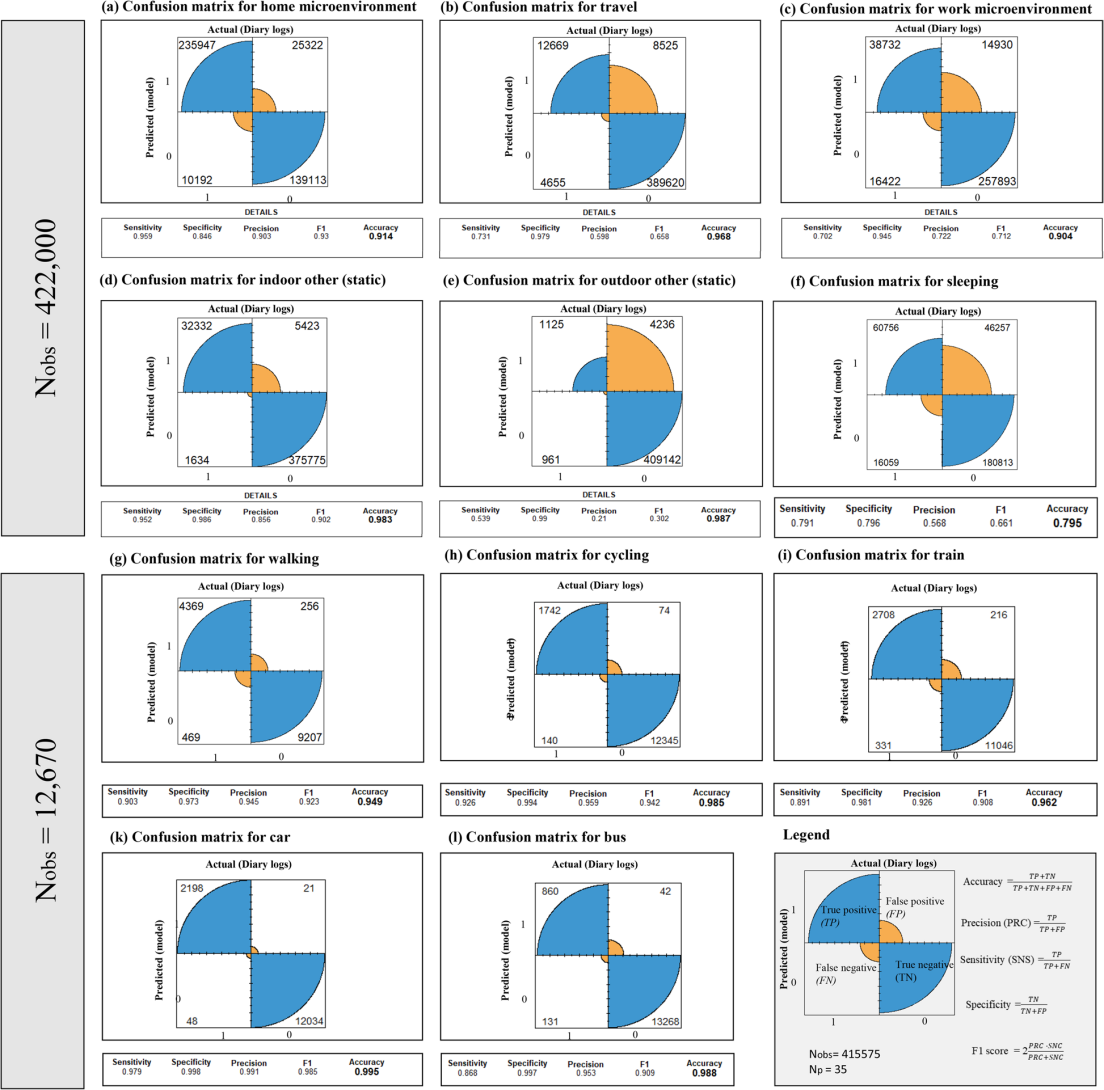
Fourfold displays of confusion matrices to visualise the performance of the space-use model. Model predictions were compared against participant logs and assigned to one of four classes represented by a quarter of a circle as shown in the legend. The size of each quarter is proportional to the counts of observations belonging to that class. Blue quarters indicate correctly classified positive and negative labels while orange quarters correspond to erroneous classifications. Quantitative evaluation metrics are displayed under each fourfold plot for each visited micro-environment. (a-f) Microenvironments and activities identified with a composite model of rule-based algorithms and spatio-temporal movement analysis. (g-l) Modes of transport classified with an RF model applied to True Positive and True Negative transit observations

The model performance was evaluated against the manual classifications. Figure 11 visualises the confusion matrices for the binary classifications of different visited microenvironments and modes of transport.

Confusion matrices represent counts from predicted and actual values. The True Negative (TN) (blue, bottom right) shows the number of negative examples classified accurately. Similarly, True Positive (TP) (blue, top left) indicates the number of positive examples classified accurately. A False Positive (FP) (orange, top right) value corresponds to the number of actual negative examples classified as positive; and a False Negative (FN) (orange, bottom left) value is the number of actual positive examples classified as negative. We examined the accuracy (the overall effectiveness of the classifier), the sensitivity (the ability of the model to identify positive labels), the specificity (the ability of the model to identify negative labels) and the precision (the proportion of positive labels that are correctly classified) of the model. We included the F1 score, which is an overall good measure that combines precision and sensitivity and is a particularly useful indicator of model performance when there is a large number of actual negatives. The range of these metrics is 0 to 1 (or 0 to 100%). The greater the value, the better is the performance of the model.

The model performed well in classifying home (Fig. 11a) with balanced FP and FN classifications (*home*: sensitivity: 96%, specificity: 85%, precision: 90%, F1: 93%, accuracy: 91%). *Other indoor static locations* (Fig. 11d) were reliably identified with a small percentage of FP (indoor: sensitivity: 95%, specificity: 99%, precision: 86%, F1: 90%, accuracy: 98%). *Sleep* and the *work* microenvironment (Fig. 11c) were classified reasonably well though only 26 out of 35 participants reported going to work (*sleep*: sensitivity: 79%, specificity: 80%, precision: 57%, F1: 66%, accuracy: 80%, *work*: sensitivity: 70%, specificity: 95%, precision: 72%, F1: 71%, accuracy: 90%).

The model overpredicted travel behaviour (Fig. 11b) and visits to outdoor static microenvironments (Fig. 11c) as shown by the relatively large number of observations classified as FP. Only 10 participants out of 35 reported a small fraction of time spent in outdoor static locations. As a result, while the accuracy and specificity for these activities were high (>96%), the precision and F1 score were lower (F1 *travel*: 66% and F1 *outdoor static*: 30%). A possible explanation is that logging short-duration trips and visits to outdoor locations might interfere with the ongoing activity and were therefore not recorded but were nevertheless detected by the model.

For this reason, periods where both the spatiotemporal-use estimator and the participant diary logs reported *travel* were retained to create a good training dataset amounting to a total of 790 trips (N$_{obs}$= 12670). The RF models had an excellent performance with sensitivity> 87%, specificity> 96%, precision>91%, accuracy>95% and F1 >91% (Fig. 11g-l).

## Qualitative evaluation of the time-activity model

Despite the overall good performance of the model in classifying static microenvironments and modes of transport, we nevertheless detected inconsistencies between manual logs and model classifications. The first part uses a representative case-study participant to illustrate such inconsistencies originating either from limitations of the model itself or errors in the manual activity logs. The second part aims to understand the implications of these inconsistencies for the overall personal exposure estimations by comparing the resulting personal concentrations in different microenvironments classified with either one of the two methods for all participants and in doing so to demonstrate how automated models such as the one presented here can enhance air pollution health studies by providing a comprehensive picture of air pollution health risks in daily life.

### Proof-of-concept for an example case-study participant

**Fig. 12:**
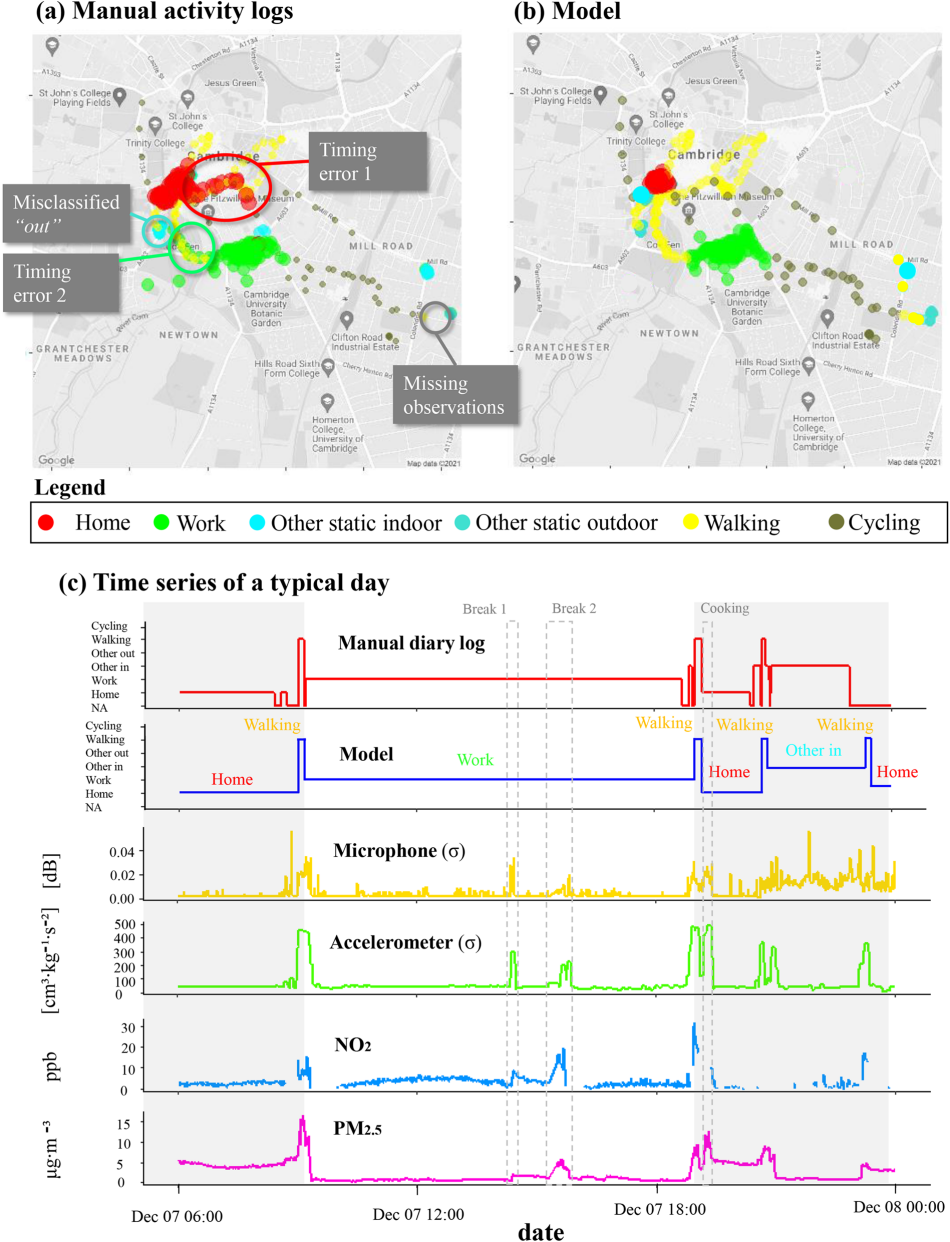
Comparison of manual logs and automated time activity model for one case study participant. Colour-coded maps illustrating visited microenvironments and modes of transport during a week of a representative participant. (a) Classifications according to the activity log. (b) Classifications according to the automated activity model. Google maps 2021. (c) Time series of the manual activity log, model classifications and selected PAM parameters for one typical day

The case study shows a representative largely sedentary office worker who commuted via cycling and walking to work and visited other indoor and outdoor microenvironments (Fig. 12). The visual inspection of the maps in Fig. 12a and b indicates that the model performance surpasses manual classification mostly due to small timing errors as the participant may have had difficulty documenting the precise time of microenvironment transitions. For example, a walking trip through the park is erroneously classified as *work* microenvironment (timing error 2, Fig. 12a). The diary was less likely to specify visits to outdoor microenvironments compared with the model (misclassified *other outdoor static*, Fig. 12a).

Figure 12c presents the time series of one typical day. The participant commuted to work on foot at around 09:00 am, stayed there until 19:00 pm and walked back home choosing a different route this time. While both methods adequately captured the participant’s time-activity patterns, the manual activity model had some missing observations and timing errors. In both trips a clear spike in all pollutants’ levels was noticed: PM$_{2.5}$ reached maximum daily concentrations during the morning walk while NO$_2$ reached maximum daily concentrations during the evening walk (Fig. 12c). The participant spent the rest of the evening cooking, resting and visiting a nearby indoor environment on foot before returning home for the night. Indoor PM$_{2.5}$ levels at home were higher than in the work environment consistent with indoor emission sources during evening cooking activities.

### Personal concentrations in visited microenvironments

**Fig. 13:**
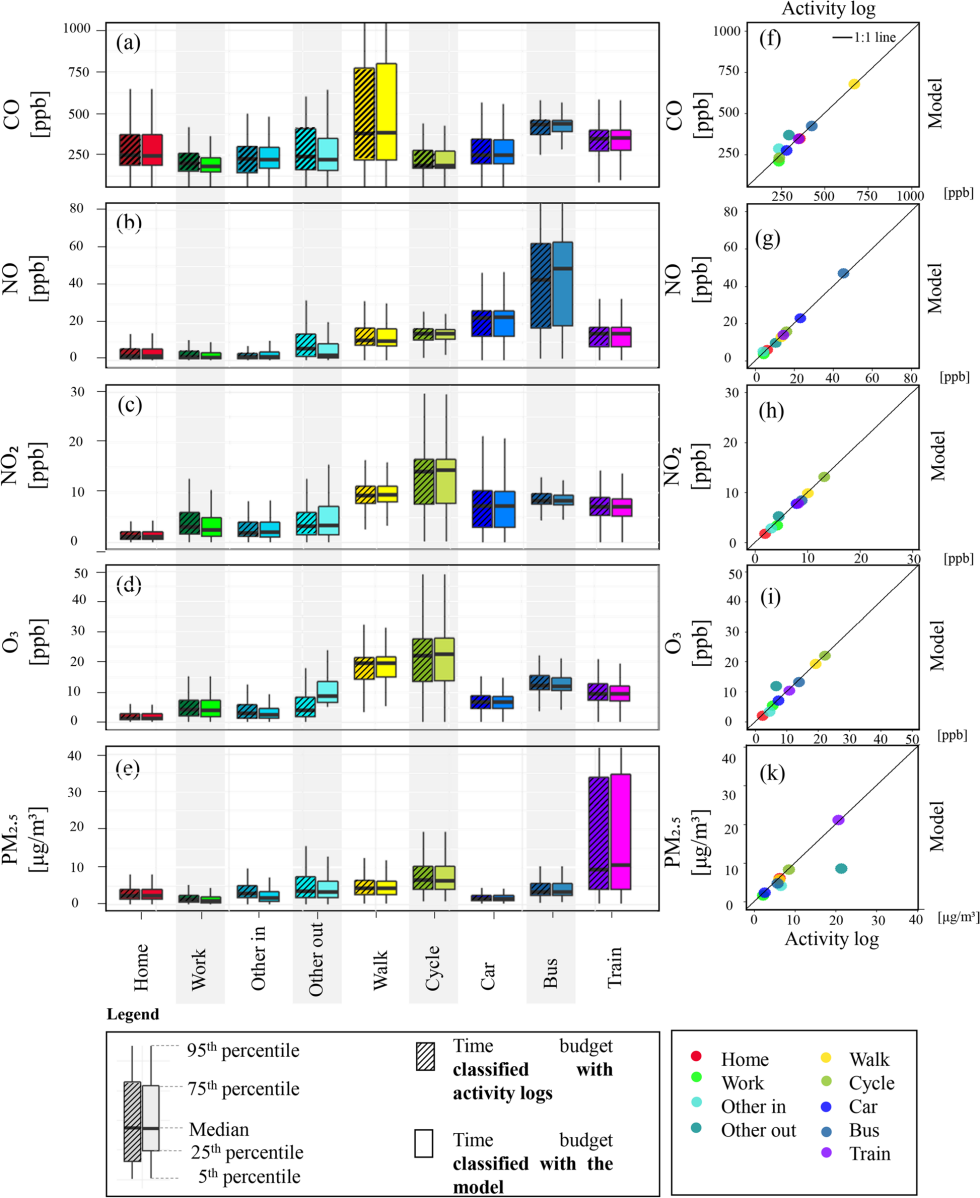
Boxplots and scatter plots of personal exposure of 35 UK participants to multiple pollutants in different microenvironments. (a-e) For each activity, the left hatched boxplot shows entries classified with participants’ activity logs and the right solid-colour boxplot with the automated model. (f-k) Mean concentrations of individual pollutants in visited microenvironments are shown in a colour-scale in scatter plots. The 1:1 line is in black

Figure 13 visualises the concentrations in different microenvironments visited by all 35 participants (N$_{obs}$ $\sim$ 422K) classified both with the manual logs and the model. The distribution of concentrations of individual pollutants in each microenvironment was visualised with boxplots (Fig. 13a). On the left-hand side, the hatched boxplot shows observations classified with the manual activity logs while the solid-colour boxplot shows observations classified with the automated model.

The corresponding scatterplots of the mean concentrations in each microenvironment are shown in Fig. 13f-k in a colour scale. Most points fall on the one-to-one line indicating that classifying microenvironments with either one of the two methods resulted in insignificant differences between estimated concentrations. *Other out* was the most poorly classified microenvironment (Fig. 11e) possibly because the whole dataset contained less than 20 participant-hours reported to be spent outside (Fig. 10a). Figure 13f-k shows that mean concentrations estimated for *other out* microenvironments had the highest deviation from the one-to-one line particularly for ozone and particulate matter (PM$_{2.5}$). The model overpredicted mean ozone concentrations compared with the activity logs. Because higher ozone levels are generally expected to be seen outdoors (Fig. 6e) due to higher levels of photochemistry, the model classifications likely outperformed the manual activity logs.

**Fig. 14:**
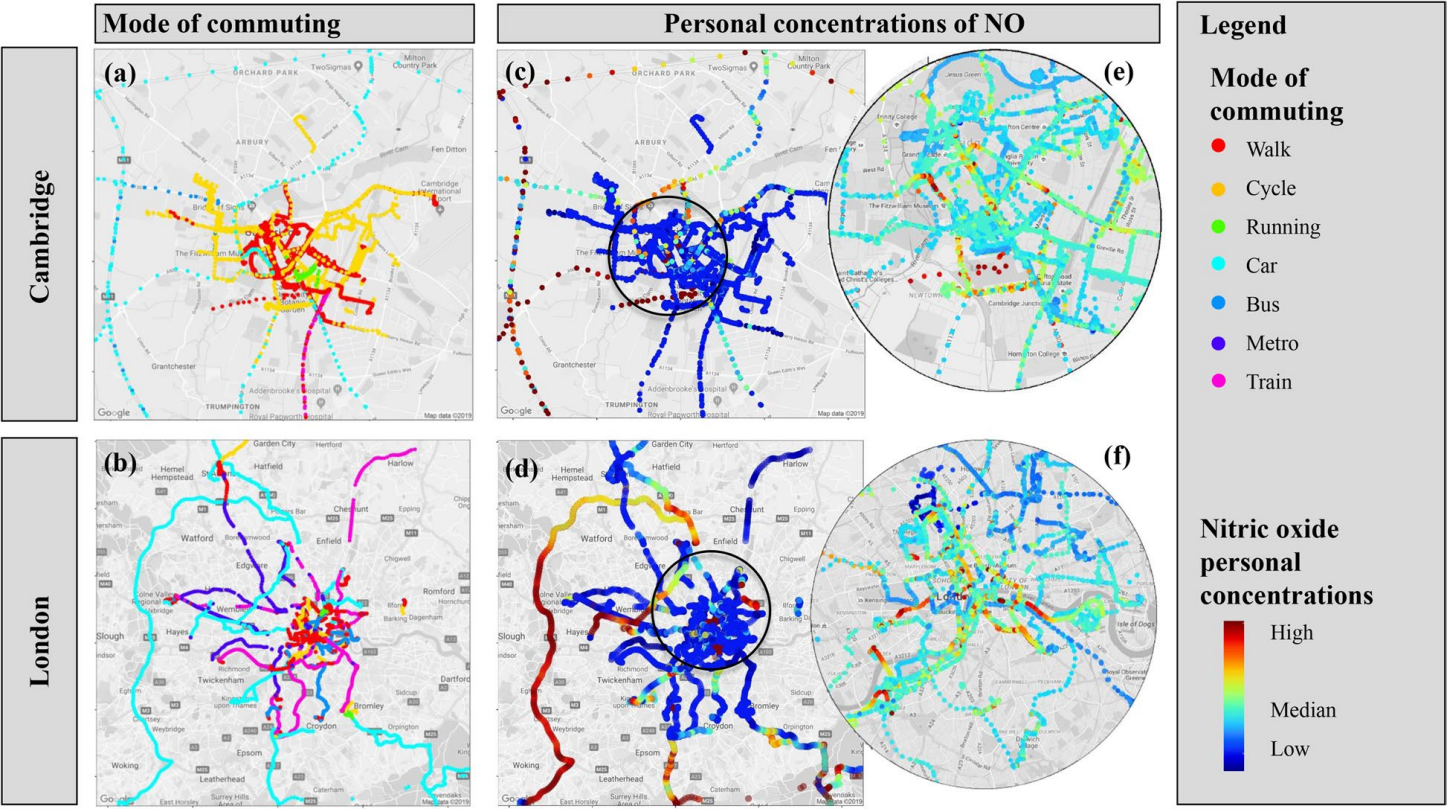
Transportation modes and relative exposure to air pollution of 35 participants plotted on maps. (a) Cambridge and (b) London visualising modes of transport (c -f) Relative exposure to pollution (in this case NO) in Cambridge and London respectively shown in a colour-scale. Map data Google 2021

Travelling in particular occupied only a small fraction of the total time budget (on average 5.2% of the participants’ time, Fig. 10a), but is a significant site of exposure (Fig. 13). Because the sample of this study is small, some caution must be applied to the interpretation and the generalisability of that finding. Participants in both cities covered large spatial distances (Fig. 14). Cambridge participants covered a smaller spatial area compared with the London participants and primarily used active modes of transport (walking, cycling). In line with previous research [61], it seems that vehicle users (car and bus) are exposed to significantly higher NO concentrations than cyclists or pedestrians (Fig. 13b), who appear to be exposed to higher NO$_2$ and O$_3$ levels(Fig. 13c-d). While this study is only a snapshot of exposure in transit, it seems that maximum air pollution levels (in this case NO) were encountered when travelling in major traffic arteries (for example M25 in the greater London area Fig. 14d) or the central bus station (Fig. 14e) and in areas where traffic is routinely static (i.e. bridges in London, Fig. 14f). Confirming previous research [62], the highest exposure to particulate matter (PM$_{2.5}$) was encountered by commuters using the train/metro system(Fig. 13e).

## Discussion

Mobile sensor deployments can provide a picture of the rapidly changing and highly granular personal concentrations in a way that has not been possible before. This paper demonstrated a methodological framework that expands the capabilities of validated sensor platforms [25] with advanced computational methods to integrate time-activity patterns in personal exposure estimations.

### Implementation of the model in different ways and programming languages

The parameters used in the time-activity model as predictors can be collected with smartphones making the method applicable more widely than with the specific sensor platforms. The model is readily extendable to include outputs from wearable biosensors in smartphones, such as heart and respiratory rate.

We employed multidisciplinary tools from the fields of movement ecology and AI and extended their use in human mobility studies to build a composite model that automatically classifies major time-activity location patterns of static spatial clusters and five modes of transport. We developed the model in R, an open-source free software environment, but equivalent algorithms can be developed in other programming languages that have similar capabilities for spatial and statistical analysis, such as Python.

### Limitations

There are certain caveats with the methodology employed to develop and evaluate the time-activity model. First, a high rate of false positives was detected for outdoor and in-transit microenvironments, although these activities generally take up a small percentage of participants’ time. We hypothesise that this is not due to limitations in the model’s accuracy, but a limitation of manual activity logs employed in the evaluation. Even the most compliant participants may have difficulty correctly documenting the precise time of microenvironment transitions, as it might interfere with the ongoing activity. Secondly, due to the increased participation burden, the sample size of 35 participants was relatively small; however, previous research on time-activity patterns and transportation mode classification has reported that a sample size of around 30 participants is adequate to provide robust estimations of activity patterns [24, 63].

### Main findings

The model had an overall good performance: the classification for static microenvironments had an F1-score for *home* of 0.93; for *work* of 0.71; for *other indoor static* of 0.9. The RF model for transportation mode classification had an excellent performance (F1 > 0.88). We found that the difference in concentrations of multiple pollutants in the nine microenvironments classified with either model or activity log was insignificant compared with the large spatial and temporal variation of personal exposure concentrations during daily life.

In line with previous research, street-level modes of commuting were associated with the highest levels of NO$_2$ and O$_3$ concentrations [61], in-vehicle trips (car and bus) were associated with marked exposure to NO [61] while the metro was associated with the highest exposure to PM [62]. These noticeable variations in concentrations between different microenvironments result in diverse personal exposures emphasising the potential for exposure misclassification when purely ecological (home location-based) exposure estimations are used in epidemiological research.

### Future work

The next step involves the application of the model on larger health panel studies [30, 31] of hundreds of participants to characterise the exposure of vulnerable subgroups of the population in diverse geographical settings. As physical activity may lead to differing doses for similar exposures, future work aims to capture total personal multi-pollutant dose in unprecedented detail addressing a major gap in air pollution epidemiology. We will further investigate whether physical activity levels may be reliable physical, psychological, social, and cognitive health indicators for elderly and chronically ill cohort participants.

More importantly, as the pollution mixture inhaled during different activities likely originates from different emission sources, it may contain different chemicals with varying potential toxicity [64]. Therefore, neglecting the activity component in air pollution dose-health relationships might lead to erroneous conclusions regarding the toxicity of air pollutants. The time activity model enables the dissagregation of total personal exposure into different microenvironment-specific exposures from diverse emission sources and chemical sinks. Together with advanced source apportionment methods of personal exposure, future work aims to explore source-specific health effects.

## Conclusions

Novel sensor technologies and computational techniques such as those demonstrated here have advantages over traditional time-activity-location diaries, which are laborious, prone to error and involve a limited number of participants. Collecting a wealth of time-activity information in unprecedented detail can increase our understanding of air pollution exposures and exposure-related behaviours that may be harmful to human health. Because individuals may have different susceptibilities to environmental exposures, together with the advancing field of *“-omics”*, this work builds towards providing comprehensive personalised advice to the individual to reduce their environmental health risks based on their unique health requirements and lifestyle.

## Supplementary Information

**Additional file 1:** **The Personal air pollution monitor and data procedures.** A brief description of the PAM sensor platform and data cleaning/feature extraction for the GPS coordinates, accelerometer and microphone readings. **Dealing with missing GPS observations.** Satellite signal loss in indoor environments is common. This section describes the rule-based algorithm developed to interpolate missing locations. **Variables evaluated for mode of transport classification.** Description of all PAM variables and extracted variables from spatio-temporal movement analysis used for RF model development. **Participant recruitment and feedback.** Descriptive summary of participants’ characteristics, recruitment timeline, example of personal exposure feedback and grouping of manual logs into main categories.

## Data Availability

The datasets generated and$\backslash$or analysed during the current study are not publicly available due to sensitive information but are available from the corresponding author on reasonable request.

## Abbreviations

AI: Artificial Intelligence
$\alpha$-NN: adaptive Nearest Neighbours
CART: Classification And Regression Trees
CO: Carbon monoxide
GIS: Geographic Information System
GPS: Global Positioning System
NO: Nitric oxide
NO$_2$: Nitrogen dioxide
O$$_3$$: Ozone
OOB: Out-Of-Bag (error)
PAM: Personal Air quality Monitor
PM: Particulate Matter
QA/QC: Quality Assurance/Quality Control
RF: Random Forest
RH: Relative Humidity
VI: Variable Importance
PAR: Perimeter-to-Area Ratio

## Footnotes

1 with the exception of the larger fraction of PM for sleeping activity

2 Geometric estimators aim to delineate the spatial extent of an individual’s movement by constructing polygons (called *hulls*) of all visited places. Probabilistic estimators create the probability density (called *utilisation distribution*) that an individual is found at a given point in space and represent the density of use of space. Widely used geometric methods are convex hull methods while the most common probabilistic methods are kernel density methods to analyse animal territory and movement [32].

3 The adaptive method specifies that the sum of the distances of all nearby points around each parent point is less than or equal to $\alpha$. Essentially, this method adjusts the size of the circles that enclose nearest neighbours based on the frequency of use of each area. In regions with more data, smaller circles can be constructed resulting in a higher resolution of space-use metrics. Because $\alpha$ is defined empirically, we used an automated method to find a suitable value for each participant [34].
