# Supplementary material for: Automated classification of time-activity-location patterns for improved estimation of personal exposure to air pollution
Source: Environ Health. 2022 Dec 9;21:125. doi: 10.1186/s12940-022-00939-8 (PMC9733291; doi:10.1186/s12940-022-00939-8)
Supplement: Supplementary file 1 — Additional file 1: The Personal air pollution monitor and data procedures. A brief description of the PAM sensor platform and data cleaning/feature extraction for the GPS coordinates, accelerometer and microphone readings. Dealing with missing GPS observations. Satellite signal loss in indoor environments is common. This section describes the rule-based algorithm developed to interpolate missing locations. Variables evaluated for mode of transport classification. Description of all PAM variables and extracted variables from spatio-temporal movement analysis used for RF model development. Participant recruitment and feedback. Descriptive summary of participants’ characteristics, recruitment timeline, example of personal exposure feedback and grouping of manual logs into main categories. [file 12940_2022_939_MOESM1_ESM.pdf]

ADDITIONAL FILES

Additional files for:

Automated classification of time-activity-location patterns for improved estimation of personal exposure to air pollution

Lia Chatzidiakou[1*], Anika Krause[1,2], Mike Kellaway[3], Yiqun Han[4], Yilin Li[1], Elizabeth Martin[1], Frank J Kelly[4], Tong Zhu[5], Benjamin Barratt[4] and Roderic L Jones[1]

*Correspondence: ec571@cam.ac.uk
[1]Yusuf Hamied Department of Chemistry, University of Cambridge, Lensfield Rd, CB2 1EW Cambridge, UK
Full list of author information is available at the end of the article

S1. The Personal air pollution monitor and data procedures

The PAM: is an autonomous unit that incorporates multiple sensors for activity, air quality and thermal conditions[1]. Following the methodology described in that paper, the raw measurements of gaseous pollutants and particulate matter were converted to physical units. The compact and lightweight design of the PAM ($\sim$ 400 g) makes the unit suitable for personal exposure assessment. The PAM was deployed in an easy-to-use carry case for protection. The time resolution of the measurements was set at 20 sec time intervals in the UK deployments[2] and 1 min in the Chinese deployment[3] resulting in a battery life on a single charge for $\sim$10 and $\sim$20 hours respectively.

GPS receiver: Detailed data on location and speed were captured using an integrated GPS unit [4] with high precision positioning. Diagnostic information of the GPS quality was collected for each spatial point including number of satellites visible, satellite fix, and horizontal dilution of precision (HDOP). Raw GPS data were not consistently reliable due to errors caused by multi-path signal reflection, loss or blocking which was primarily observed indoors. The multi-path problem occurred mainly in urban areas where tall buildings and structures reflect satellite signals many times before they reach a GPS device [2] leading to GPS coordinate errors. Such errors were identified and removed when the speed derived from the Euclidean distance between two consecutive points exceeded 170 m/s or when three successive points formed a linear segment (indicating no change of direction).

Accelerometer signal representing vector magnitude: The signal representing the vector magnitude of the accelerometer (svm) was calculated from the triaxial signal components x , $y$and z as:

$$svm = \sqrt{x^2 + y^2 + z^2}$$

Accelerometer and microphone feature extraction: Readings of the triaxial accelerometer and microphone were recorded at 100 Hz. In order to reduce the data transmission, feature extraction was performed in the ARM micro-processor of the PAM. The raw time-series data of the accelerometer and the microphone were grouped into 20-second non-overlapping segments. Descriptive statistics, such as minimum, maximum and mean, were

extracted for each of these windows. Additionally, four threshold values were set that correspond to sedentary, light, moderate and vigorous intensity levels of physical activity. Crossings and duration of the signal magnitude vector above these thresholds were counted for each 20-sec window.

S2. Classifying observations into microenvironments when GPS coordinates were missing

Satellite signal loss in indoor environments is common. In our dataset approximately $\sim$ 40% of the GPS coordinates were missing. To classify these observations, a rule-based algorithm was developed (Figure A1). The algorithm firstly aimed to detect whether there was a change from the previous to the last known location. For example, signal loss was primarily observed during the night-time when the PAM was left inactive for extended time periods. In that case, static location remained unchanged before and after satellite signal loss and the observations within that time window would be classified as "home". This scenario was the most common accounting for about 70% of the missing data. Additionally, travelling in the underground metro system or in urban areas with tall buildings (for example, working in an office in central London) also resulted in GPS signal loss. In this scenario, separating static from in-transit classifications within that time window would require accelerometry information.

Figure A1 Rule-based algorithm to fill in missing GPS observations.

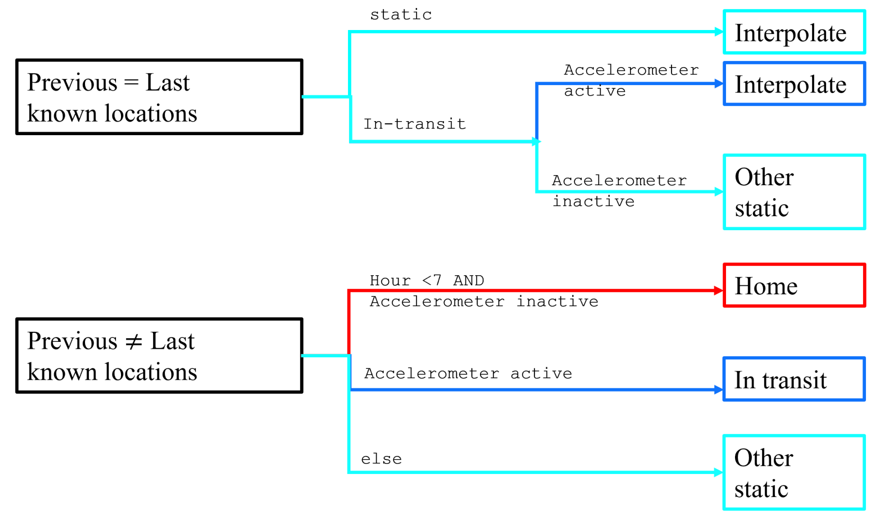

S3. Variables evaluated for mode of transport classification with Random Forest models

The periods classified as *in transit* were classified into five modes of transportation. Variable selection for the classification was implemented using RF in the `VSURF` package[5] in R. We included 31 variables collected with the PAM as shown in the table A1 below. We also included metrics extracted from the spatio-temporal analysis summarised in Table A2.

Table A1 31 variables collected with the PAM

Variable	Description	Source: PAM sensor
hour	local hour	GPS
longitude		GPS
latitude		GPS
altitude		GPS
gps sats	N visible satellites	GPS
gps hdop	horizontal dilution of precision	GPS
gps fix	position solution	GPS
mic mean (min, max, σ)	Mean (min, max, σ) value of 1 min window from 100 Hz	microphone
mic c χ	Counts above four thresholds χ in 1 min window from 100 Hz	microphone
mic d χ	Duration above four thresholds χ in 1 min window from 100 Hz	microphone
svm[1] mean (min, max, σ)	Mean (min, max, σ) value of 1 min window from 100 Hz	Tri-axial accelerometer
svm c ψ	Counts above four thresholds ψ in 1 min window from 100 Hz	Tri-axial accelerometer
svm d ψ	duration above four thresholds ψ in 1 min window from 100 Hz	Tri-axial accelerometer

svm **signal of vector magnitude**

Table A2 19 variables from spatio-temporal movement analysis

Variable	Description	Source: `R` package
nnn	closest neighbours of each point	`TLoCoH`
area	area of the isopleth the point belongs to	`TLoCoH`
perim	perimeter of isopleth	`TLoCoH`
tspan	timespan	`TLoCoH`
nep	number of enclosed points in isopleth	`TLoCoH`
scg.enc.mean (σ)	average (σ) speed of all points enclosed in isopleth	`TLoCoH`
scg.nn.mean (σ)	average (σ) speed of all points identified as nearest neighbors	`TLoCoH`
elongation	eccentricity of ellipse enclosing hull	`TLoCoH`
nsv	Visitation rate	`TLoCoH`
mnlv	Duration of visit	`TLoCoH`
abs.angl	the angle between each move and the x axis	`AdehabitatLT`
rel.angl	the turning angles between successive moves	`AdehabitatLT`
R2n	the squared net displacement between the current relocation and the first relocation of the trajectory	`AdehabitatLT`
comevent	Commuting event (trajectory)	`AdehabitatLT`
burst	Segment of trajectory	`AdehabitatLT`
dist euc	Euclidean distance between two successive points	
dist	length of move	`AdehabitatLT`

S4. Participant recruitment and feedback

Figure A2 Participant recruitment for the evaluation of the time activity model. Each line represents the participation period of an individual. In total, 13 individuals residing in Cambridge (black) and 24 in London (blue) were recruited. The study took place from July to December 2015.

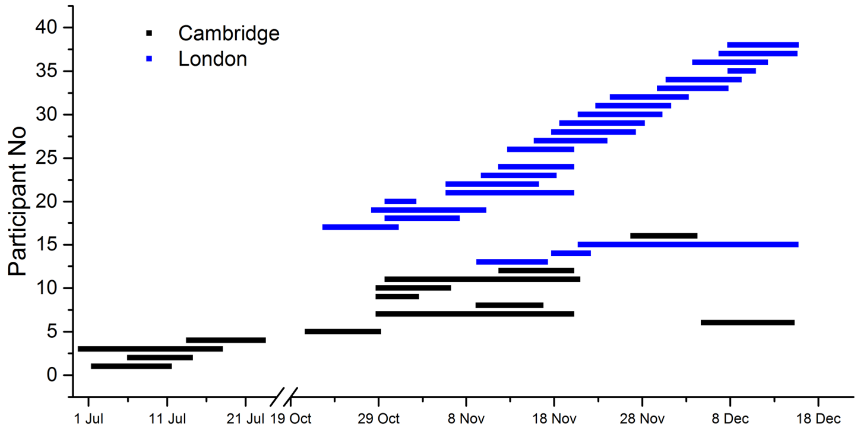

Table A3 Personal and socio-economic information of participants

**Participants**	N= 37
**Residence**	
London	24
Cambridge	13
**Age**	
18-22	2
23-30	13
31-40	10
41-50	4
51-60	3
61-65	2
Unknown	3
**Gender**	
Female	20
Male	17
**Education**	
Secondary school	1
Further education (A-Level or similar)	1
Higher Education (degree)	32
Unknown	3

Overall, the participants reported 665 time-activity entries. These entries were assigned to a main theme (for example, office work: writing, reading, coding etc) which was grouped to two core categories: *location* and *activity* as shown in Table A4 below.

Figure A3 Personalised participant feedback produced as a compensation for their contribution in the study. Page 1: Short description of the aims of the study and a map with relative exposure to NO as they went about their day. Page 2: Advice to reduce personal exposure in daily life (top) and relative exposure over a week to multiple pollutants. Page 3: Time-series of multiple pollutants and activity parameters of a typical participant day.

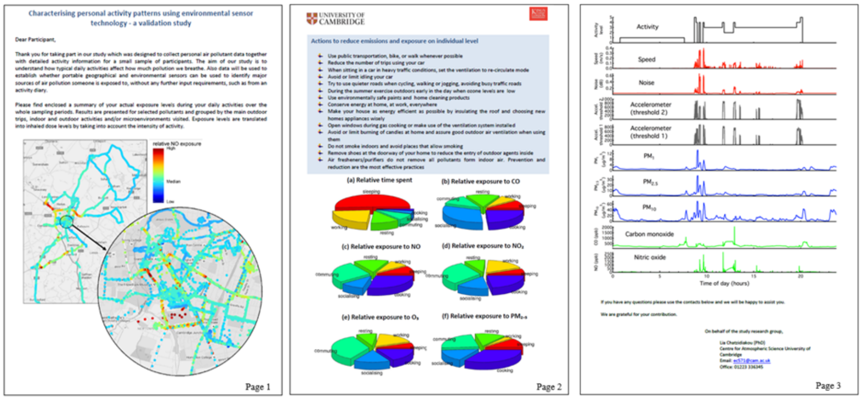

Table A4 Location and activity classifications derived from the semi-structured manual activity logs. Bold indicate classifications that this paper focuses on with more detailed work in the future on cooking activities and indoor emission sources characterisation.

Location	Activity	Main themes from reported activities
	cooking	baking, frying, grilling, stove
**home**	resting	bedroom, candle, fireplace, meditating, online, reading/studying, resting, TV, gaming
	personal care	housework, eating, shower, hairdryer
	**sleeping**	
**work**	work	lab, meeting, office work, lecture, break
**in transit**	**walking**	
	running	
	**cycling**	
	**train/metro**	including overground, station
	**car**	including taxi
	**bus**	including bus stop
**other locations**	socialising	cinema, coffee, friends, pub, restaurant
	other (high intensity)	childcare, gardening, shopping, sports
	other activities	library, hospital visit, dentist, church, singing, smoking

Author details
[1]Yusuf Hamied Department of Chemistry, University of Cambridge, Lensfield Rd, CB2 1EW Cambridge, UK. [2]Institute for Chemistry, University of Potsdam, Karl-Liebknecht-Straße 24-25, 14476 Potsdam, Germany. [3]Atmospheric Sensors Ltd, SG19 3SH Bedfordshire, UK. [4]Environmental Research Group, MRC Centre for Environment and Health, Imperial College London, W12 0BZ London, UK. [5]BIC-ESAT and SKL-ESPC, College of Environmental Sciences and Engineering, Center for Environment and Health, Peking University, 100871 Beijing, China.

References

1. Chatzidiakou, L., Krause, A., Popoola, O.A., Di Antonio, A., Kellaway, M., Han, Y., Squires, F.A., Wang, T., Zhang, H., Wang, Q., *et al.*: Characterising low-cost sensors in highly portable platforms to quantify personal exposure in diverse environments. Atmospheric measurement techniques **12**(8), 4643–4657 (2019)
2. Moore, E., Chatzidiakou, L., Jones, R.L., Smeeth, L., Beevers, S., Kelly, F.J., Quint, J.K., Barratt, B.: Linking e-health records, patient-reported symptoms and environmental exposure data to characterise and model copd exacerbations: protocol for the cope study. BMJ open **6**(7), 011330 (2016)
3. Han, Y., Chen, W., Chatzidiakou, L., Krause, A., Yan, L., Zhang, H., Chan, Q., Barratt, B., Jones, R., Liu, J., *et al.*: Effects of air pollution on cardiopulmonary disease in urban and peri-urban residents in beijing: protocol for the airless study. Atmospheric Chemistry and Physics **20**(24), 15775–15792 (2020)
4. u-blox GNSS product overview. Technical report (2019). www.u-blox.com/guided-product-selector
5. Genuer, R., Poggi, J.-M., Tuleau-Malot, C.: Variable selection using random forests. Pattern recognition letters **31**(14), 2225–2236 (2010)
